# IL-23 supports host defense against systemic *Candida albicans* infection by ensuring myeloid cell survival

**DOI:** 10.1371/journal.ppat.1008115

**Published:** 2019-12-30

**Authors:** Selim Nur, Florian Sparber, Christina Lemberg, Eva Guiducci, Tiziano A. Schweizer, Pascale Zwicky, Burkhard Becher, Salomé LeibundGut-Landmann

**Affiliations:** 1 Section of Immunology, Vetsuisse Faculty, University of Zürich, Zürich, Switzerland; 2 Institute of Experimental Immunology, University of Zürich, Zürich, Switzerland; Tulane University School of Medicine, UNITED STATES

## Abstract

The opportunistic fungal pathogen *Candida albicans* can cause invasive infections in susceptible hosts and the innate immune system, in particular myeloid cell-mediated immunity, is critical for rapid immune protection and host survival during systemic candidiasis. Using a mouse model of the human disease, we identified a novel role of IL-23 in antifungal defense. IL-23-deficient mice are highly susceptible to systemic infection with *C*. *albicans*. We found that this results from a drastic reduction in all subsets of myeloid cells in the infected kidney, which in turn leads to rapid fungal overgrowth and renal tissue injury. The loss in myeloid cells is not due to a defect in emergency myelopoiesis or the recruitment of newly generated cells to the site of infection but, rather, is a consequence of impaired survival of myeloid cells at the site of infection. In fact, the absence of a functional IL-23 pathway causes massive myeloid cell apoptosis upon *C*. *albicans* infection. Importantly, IL-23 protects myeloid cells from apoptosis independently of the IL-23-IL-17 immune axis and independently of lymphocytes and innate lymphoid cells. Instead, our results suggest that IL-23 acts in a partially autocrine but not cell-intrinsic manner within the myeloid compartment to promote host protection from systemic candidiasis. Collectively, our data highlight an unprecedented and non-canonical role of IL-23 in securing survival of myeloid cells, which is key for maintaining sufficient numbers of cells at the site of infection to ensure efficient host protection.

## Author summary

Linked to advances in medical technology and the resulting increase in the number of intensive care patients, nosocomial infections with *Candida albicans* are on the rise. In patients suffering from invasive candidiasis the innate immune response is typically severely impaired. Strengthening the innate immune system has become a promising approach complementing the use of antifungal drugs. Our findings identify an unexpected and IL-17-independent role of IL-23 that prevents rapid death of myeloid cells during systemic candidiasis and thereby promotes optimal protection from disease. As such, IL-23 represents an important new piece in the puzzle of the finely tuned network of cytokines that regulates the innate immune response to fungal infection. Our results contribute to a better understanding of myeloid cell regulation during infection and thereby open new perspectives for future immunotherapeutic applications that may improve patient outcome.

## Introduction

*Candida albicans* has emerged as a clinically important opportunistic fungal pathogen causing disease mainly in immunocompromised individuals. In severe cases, *C*. *albicans* disseminates from colonized mucosal surfaces or the skin and gives rise to systemic infections [1]. Rapid fungal spread from the circulation to visceral organs may be followed by overt fungal growth and organ failure, if uncontrolled. Systemic infections with *C*. *albicans* are associated with poor prognosis and a high mortality rate [2]. According to conservative estimates, invasive candidiasis affects more than 250,000 people worldwide annually and is the cause of over 50,000 deaths per year [3].

The innate immune system is vital to mount a rapid and efficient response to systemic infection with *C*. *albicans*. The efficacy of the immediate response during the first hours to days after fungal entry into the bloodstream determines whether fungal dissemination can be contained and fungal growth limited or not. Myeloid cells figure prominently in this decisive response at the onset of infection. As such, defects in the myeloid compartment, e.g. neutropenia, are well-established high-risk factors for systemic *Candida* infections in humans [4,5]. In the murine intravenous model of systemic candidiasis, the lack of neutrophils results in a dramatic increase in fungal control and rapid death of the infected animal [6]. Besides the key role of neutrophils, other myeloid cells also critically contribute to fungal control. Depletion of CCR2^+^ monocytes in mice leads to rapid uncontrolled growth of *C*. *albicans* in the kidneys and brain, highlighting an essential protective function of these cells at the onset of systemic candidiasis [7]. Moreover, tissue-resident renal macrophages are also critical for early defense, as *CX3CR1*^*-/-*^ mice with defects in this cell type display impaired host survival due to an elevated fungal burden in the kidney and *C*. *albicans*-induced renal failure [8].

IL-23 expression is strongly induced in response to *C*. *albicans* via the C-type lectin pathway [9,10] and is best known for regulating IL-17 production by T cells and innate lymphoid cells [11], which is of particular relevance for antifungal immunity at epithelial barriers [12]. IL-17 also contributes to protection from disseminated candidiasis [13–15]. Importantly, a growing body of work suggests that IL-23 possesses functions that exceed regulation of IL-17 immunity. For instance, IL-23 has been demonstrated to drive expression of GM-CSF, which is essential for the induction of neuroinflammation in experimental autoimmune encephalomyelitis [16,17]. Furthermore, IL-23 has been shown to induce dermal inflammation and acanthosis in an IL-17-independent manner [18,19]. IL-23-mediated effects that are independent of T cells have also been reported [20–24].

Numerous studies in humans and mice have brought important insights into the recruitment of myeloid cells from their reservoirs to the site of infection, the activation of their effector functions and their constant replenishment to ensure optimal protection from *C*. *albicans*. Yet, little attention has been paid to the question of how myeloid cell viability is maintained during infection and how myeloid cells are eliminated once the infectious threat is overcome. Here we describe an unprecedented novel role of IL-23 in promoting myeloid cell viability during systemic candidiasis. We show that IL-23 critically contributes to enhanced protection during the critical phase at the onset of infection by preventing apoptosis of neutrophils, monocytes and other mononuclear phagocytes in the infected kidney, and to a lesser extent in the brain. Our results reveal a hitherto unappreciated IL-17-independent role for IL-23 in control of myeloid cell viability during disseminated candididiasis that may be of relevance for the understanding of human disease.

## Results

### IL-23 signaling is essential for fungal control and host protection from systemic candidiasis

**Fig 1:**
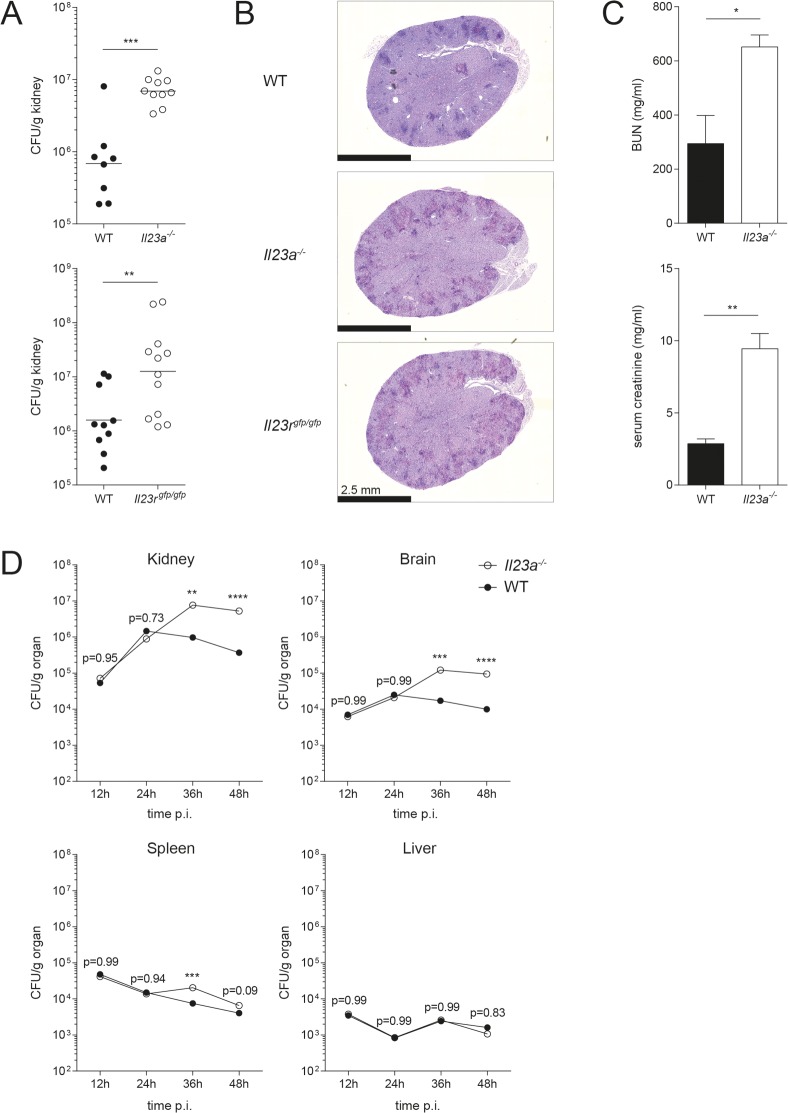
IL-23- and IL-23R-deficient mice are highly susceptible to systemic *C*. *albicans* infection. *Il23a*^*-/-*^ and WT mice were infected with 2x10^5^ CFU *C*. *albicans*. *Il23r*^gfp/gfp^ mice and their respective controls were infected with 3x10^5^ CFU *C*. *albicans*. (A) Kidney fungal burden was determined at 48h post infection. Each dot represents one animal. The mean of each group is indicated. Graphs show pooled data from three independent experiments. (B) Transversal kidney sections were stained with PAS reagent 48h post infection. Representative images are shown. Scale bar = 2.5 mm. (C) Blood urea nitrogen (BUN) and creatinine levels were measured in the serum of the indicated groups 48h post infection. Bars are the mean with standard deviation (SD) of each group with n = 3. Data are representative of two independent experiments. (D) Fungal burden of each organ was determined at the indicated time points. Data shown are the mean of three animals. Statistics were calculated using unpaired Student’s t-Test (A, C) or two-way ANOVA (D) as appropriate. *p<0.05, **p<0.01, ***p<0.001, ****p<0.0001.

To study the role of IL-23 in innate antifungal immunity, we infected *Il23a*^*-/-*^ mice with *C*. *albicans* via the systemic route. In accordance with [10], we found that IL-23-deficient mice displayed highly increased fungal burden in the kidney, the major target organ of *C*. *albicans* in this model, 48h post infection compared to their wildtype counterparts **(Fig 1A).** Systemic *C*. *albicans* infection of *Il23r*^gfp/gfp^ mice, which equal IL-23R-deficient mice if the IL-23R-GFP knock-in allele is bred to homozygosity [25], mirrored infection of IL-23 cytokine-deficient mice with a massively increased fungal burden compared to their WT counterparts **(Fig 1A)**. Along with the high fungal burden, *Il23a*^*-/-*^ and *Il23r*^gfp/gfp^ mice, but not their wildtype counterparts, also displayed signs of pain and severe distress at 48h post infection, indicative of severe systemic disease. Extensive dissemination of *C*. *albicans* was observed throughout the renal cortex of Periodic acid–Schiff (PAS)-stained kidney sections of mice deficient in IL-23 production or IL-23 signaling **(Fig 1B)**. As *C*. *albicans* can cause substantial renal tissue injury [26], we quantified blood urea nitrogen (BUN) and serum creatinine, two markers of kidney function, in the serum of infected mice. Indeed, we found highly elevated levels of both in the serum of IL-23-deficient mice compared to WT controls **(Fig 1C)**.

Given the very rapid and drastic differences in kidney fungal control between WT and IL-23 signaling-deficient mice, we were interested in the temporal and spatial dynamics of fungal spread. To that aim, we assessed every 12h over the first 48h of infection the fungal colonization levels in diverse organs known to be targeted by *C*. *albicans*. In agreement with the literature [27], we found that fungal burden declined rapidly in spleen and liver, while it remained high or even further increased in kidney and brain **(Fig 1D)**. No differences were observed between WT and *Il23a*^*-/-*^ mice during the initial 24h post infection. However, starting from 36h post infection, fungal loads were significantly increased in the kidney of IL-23-deficient mice compared to WT controls, confirming our previous results **(Fig 1D)**. Likewise, fungal outgrowth was observed in the brain in absence of IL-23 at 36h and 48h post infection, while in spleen and liver the fungus was controlled to a similar extent in WT and *Il23a*^*-/-*^ mice **(Fig 1D)**. Due to ethical considerations, we did not extend our analysis to time points beyond 48h post infection as morbidity of IL-23 pathway-deficient mice rapidly increased thereafter. In conclusion, our data suggest that IL-23 signaling is fundamental for fungal control and host protection from systemic candidiasis.

### IL-23 deficiency results in a loss of myeloid, but not lymphoid cells upon systemic *C*. *albicans* infection

**Fig 2:**
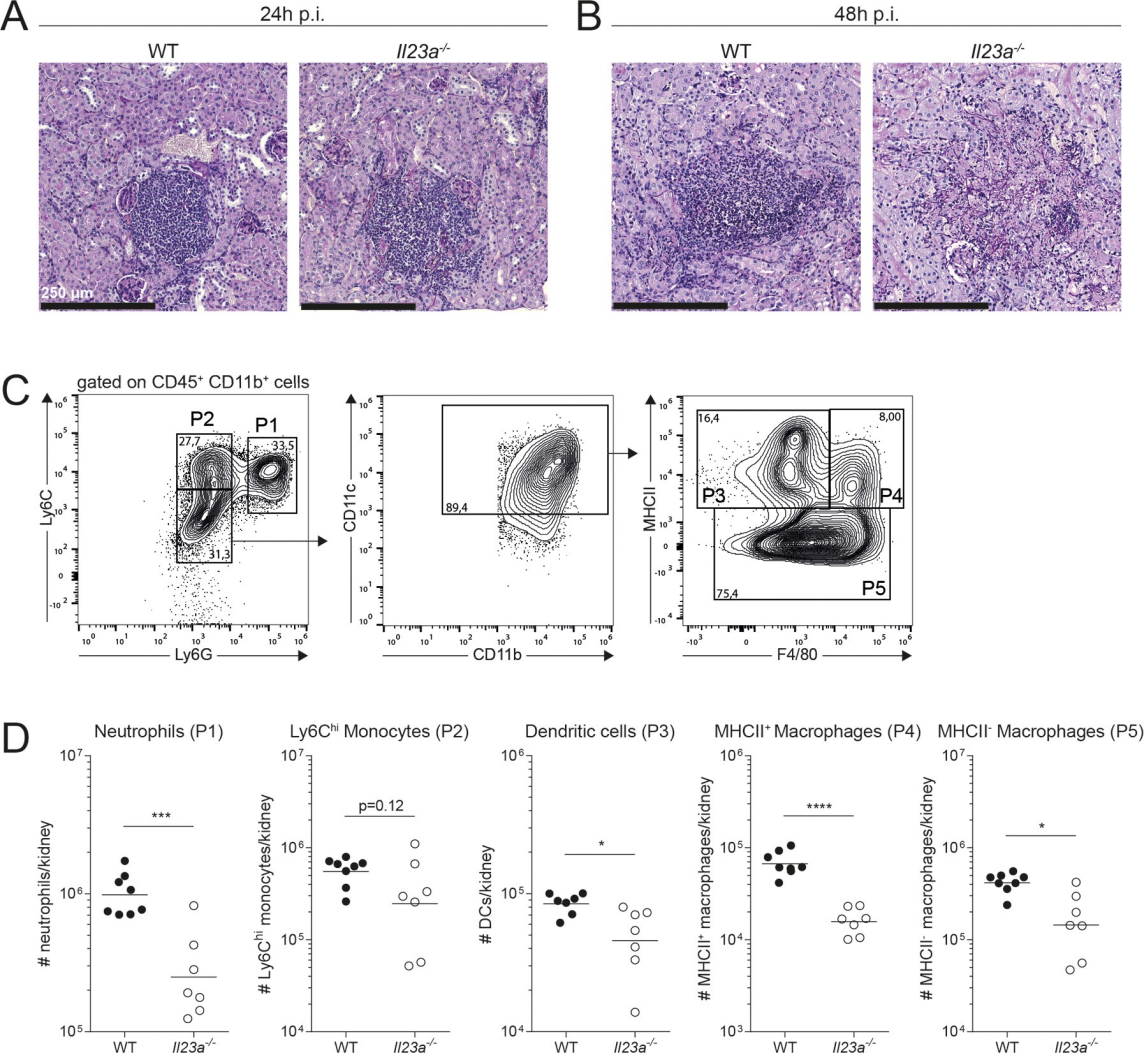
Loss of myeloid cells in the kidney of *C*. *albicans*-infected *Il23a*^*-/-*^ mice. WT and *Il23a*^*-/-*^ mice were infected intravenously with 2x10^5^ CFU *C*. *albicans*. (A-B) Transversal kidney sections were stained with PAS reagent 24h (A) or 48h post infection (B). Representative images are shown. Scale bar = 250 μm. (C-D) Myeloid cell populations in the kidney were quantified by flow cytometry 48h post infection. Representative FACS plots in (C) show the gating strategy for kidney myeloid cells. Neutrophils (P1), Ly6C^hi^ monocytes (P2), DCs (P3) and macrophages (P4 and P5) were defined as indicated. Summary graphs in (D) show the absolute numbers of each cell population per kidney. Each dot represents one animal and the mean of each group is indicated. Data are pooled from two independent experiments. Statistics were calculated using unpaired Student’s t-Test. *p<0.05, **p<0.01, ***p<0.001, ****p<0.0001.

Given the acute onset and the rapid course of disease in experimentally infected mice, the host response to systemic candidiasis predominantly relies on the innate immune system. In accordance with [27], foci of *C*. *albicans* were rapidly surrounded by dense inflammatory cell clusters in the infected kidney **(Fig 2A)** that predominantly consisted of neutrophils and mononuclear phagocytes. The situation was comparable in WT and *Il23a*^*-/-*^ mice with similar numbers of neutrophils and Ly6C^hi^ monocytes in both genotypes at 24h post infection **(Fig 2A and S1A Fig)**. Next, we examined tissue sections at 48h post infection, the time point when the biggest differences in kidney fungal load were observed between *Il23a*^*-/-*^ and WT mice **(Fig 1D)**. Despite the very high fungal burden in IL-23-deficient mice at this time point, we found to our surprise that the inflammatory infiltrates were largely absent from *C*. *albicans* foci and throughout the kidney of *Il23a*^*-/-*^ mice at 48h post infection, while dense inflammatory cell clusters further accumulated in the infected kidney of WT mice over the same period of time **(Fig 2B)**. We thus hypothesized that the disappearance of myeloid cells from the infected kidney might be the reason for the inferior fungal control in IL-23-deficient mice. Neutrophils [6] and monocytes [7], which get rapidly recruited to the infected kidney during systemic candidiasis [27], as well as macrophages [8,28] and dendritic cells [10] play a key role in innate antifungal immunity against *C*. *albicans*. By flow cytometry, we identified Ly6G^+^Ly6C^int^ neutrophils, Ly6C^hi^Ly6G^-^ monocytes, CD11c^+^F4/80^+^MHCII^+^ and MHCII^-^ macrophages and CD11c^+^MHCII^+^F4/80^-^ dendritic cells (DCs) in the infected kidney **(Fig 2C).** At 48h post infection, the time point we observed the biggest difference in fungal load between the two genotypes, all these myeloid cell populations were reduced in number in the kidney of *Il23a*^*-/-*^ mice compared to WT controls **(Fig 2D)**. To get a more complete picture, we also quantified lymphoid cell populations, more specifically CD3ε^+^TCRβ^+^ T cells, CD3ε^+^TCRγδ^+^ γδ T cells, CD3ε^-^NK1.1^-^CD90^+^ ILCs and NK1.1^+^CD3ε^-^ NK cells in the infected kidney **(S1B Fig)**. Of note, none of these populations were significantly different between infected WT and *Il23a*^*-/-*^ mice **(S1C Fig)**. Collectively, we found a drastic and selective loss of myeloid but not lymphoid cells in the kidney of *Il23a*^*-/-*^ mice compared to WT controls with the kinetics of the loss in myeloid cells paralleling the loss of fungal control at 48h post infection, but not yet at 24h post infection when fungal loads were still comparable between the two genotypes. Our data therefore suggest that the decline in myeloid cells may be accountable for uncontrolled kidney fungal burden in IL-23-deficient mice. Following the observation that fungal loads were also strongly elevated in the brain of *Il23a*^*-/-*^ mice **(Fig 1D)**, we also quantified myeloid cell populations in this organ. Neutrophils in the brain were strongly reduced in *Il23a*^*-/-*^ compared to WT mice at 48h post infection as in the kidney. The effect did not extend to all myeloid subsets with Ly6C^hi^ monocytes and Ly6C^lo^ myeloid cells, which mainly comprise CD45^lo^CD11b^+^ microglia [29], remaining largely unaffected (**S1D and S1E Fig)**. This discrepancy between kidney and brain may be explained by differences in the nature of the myeloid subsets and/or tissue-specific differences in the mechanisms of disease between these two organs.

### IL-23 deficiency does not compromise *de novo* synthesis and trafficking of myeloid cells

**Fig 3:**
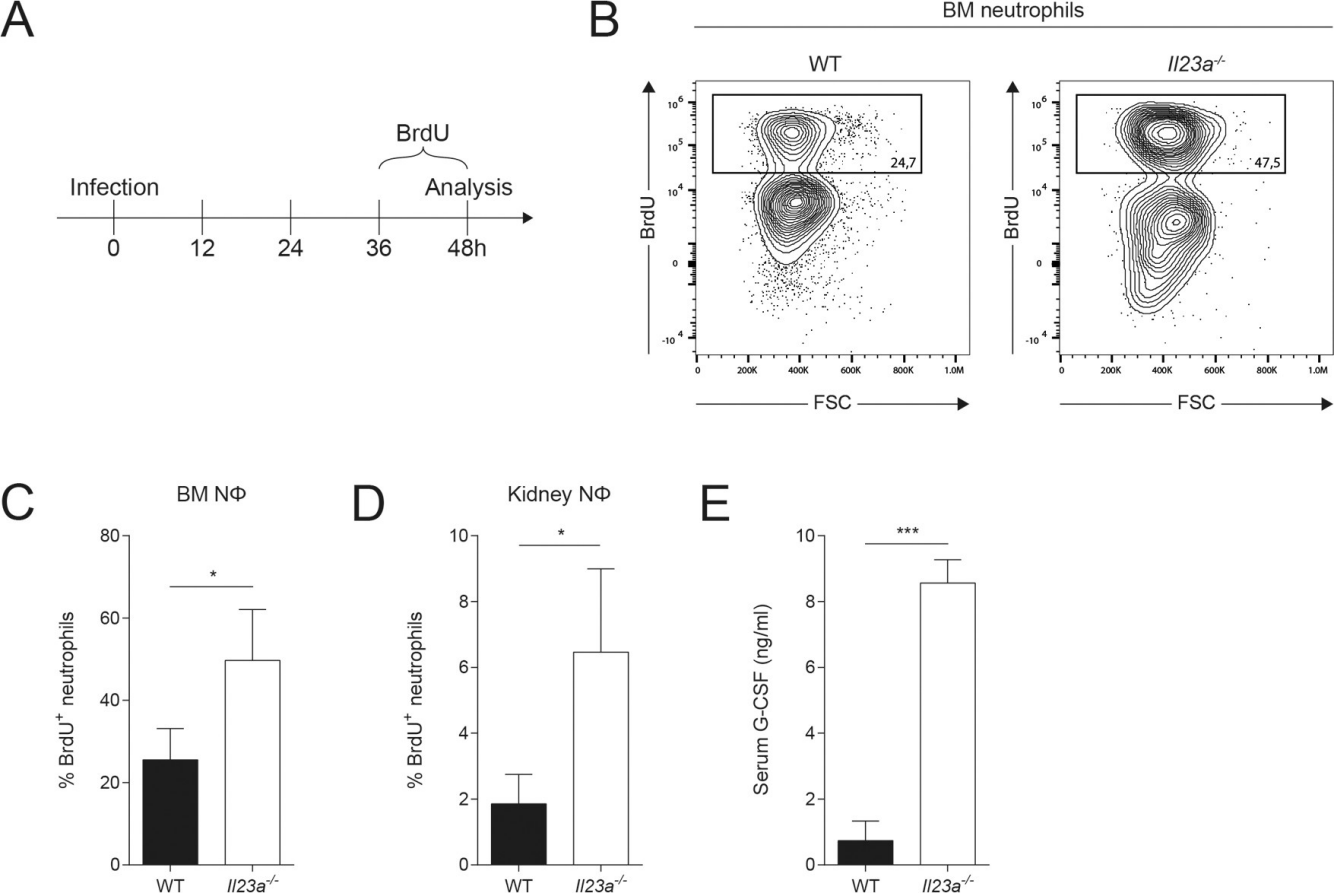
Emergency granulopoiesis and neutrophil supply is not impaired in *Il23a*^*-/-*^ mice. WT and *Il23a*^*-/-*^ mice were infected intravenously with 2x10^5^ CFU *C*. *albicans* and treated with BrdU for the last 12h prior to analysis of neutrophil proliferation by flow cytometry at 48h post infection. (A) Schematic outline of the experimental setup. (B) Representative FACS plots show the gating strategy for identifying BrdU^+^ neutrophils in the BM. Neutrophils were defined as shown in Fig 2C. (C, D) Summary graphs show percentage of BrdU^+^ cells in the total population of BM (C) or kidney neutrophils (D). (E) G-CSF levels in the serum were quantified by ELISA at 48h post infection. In C-E, bars are the mean + SD of each group with n = 3. Data are representative of two independent experiments. Statistics were calculated using unpaired Student’s t-Test. *p<0.05, **p<0.01, ***p<0.001.

Myeloid cells and in particular the circulating subsets are short-lived, especially after pathogen encounter and exertion of their effector functions. Thus, tightly regulated *de novo* myelopoiesis is a prerequisite for maintaining a continuous supply of the cells during periods of increased demand. We therefore hypothesized that the decline in myeloid cells at the site of infection between 36h and 48h post infection in absence of a functional IL-23 pathway could be the result of impaired bone marrow (BM) activity. This would fit a scenario in which early supply of cells would be granted by the pre-existing pool of neutrophils and monocytes in the circulation and BM over the first 24h of infection, but inadequate *de novo* production would then result in insufficient supply to the site of infection at later time points. We therefore examined emergency granulopoiesis by an *in vivo* labeling approach with bromodeoxyuridine (BrdU), a synthetic thymidine analogon that is incorporated into newly synthesized DNA. WT and *Il23a*^*-/-*^ mice were treated with BrdU during the last 12h before analysis at 48h post infection **(Fig 3A)**. To our surprise, we found a significantly higher proportion of BrdU^+^ neutrophils in the BM of IL-23-deficient mice compared to WT controls at 48h post infection, actually indicating increased rather than decreased emergency granulopoiesis in absence of IL-23 **(Fig 3B and 3C)**.

We next wondered whether trafficking of neutrophils from the BM and blood to the site of infection was impaired. To this aim, we assessed whether BrdU-labeled *de novo* generated neutrophils arrived in the kidney. We again found a significantly increased proportion of BrdU^+^ neutrophils in the kidney **(Fig 3D)**, indicating that there was not only an increased turnover but also superior supply of neutrophils to the site of infection in absence of functional IL-23 signaling compared to WT controls. In support of these results, we detected increased levels of G-CSF, a hallmark granulopoiesis-inducing cytokine, in the serum of *Il23a*^*-/-*^ mice compared to their WT counterparts at 48h post infection **(Fig 3E)**. We also assessed the rate of granulopoiesis at 24h post infection, when fungal burden was still comparable in WT and *Il23a*^*-/-*^ mice by providing BrdU between 12h and 24h post infection **(S2A Fig)**. Under these conditions, we found comparable proportions of BrdU^+^ neutrophils in the BM and kidney of both genotypes **(S2B Fig)**, indicating that enhanced granulopoiesis follows the decline in myeloid cell numbers in the infected kidney. Together, our data indicate that *de novo* granulopoiesis and trafficking of neutrophils was not impaired in IL-23-deficient mice and was even superior to that in WT mice, yet apparently insufficient to confer fungal control and host protection during systemic candidiasis.

### The loss of myeloid cells in the *C*. *albicans*-infected kidney of *Il23a*^*-/-*^ mice results from their impaired survival

**Fig 4:**
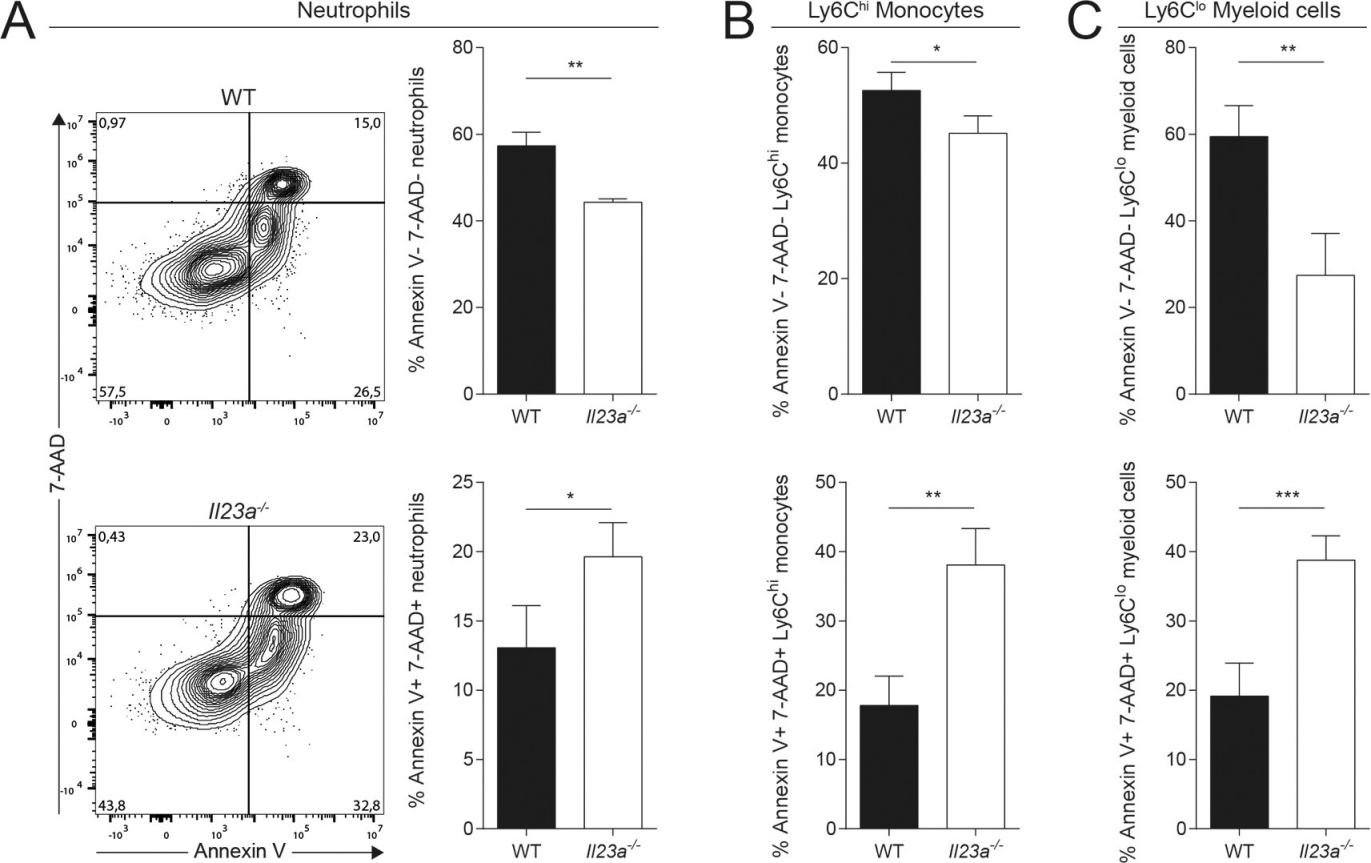
Viability of myeloid cells is impaired in *Il23a*^*-/-*^ mice upon infection. WT and *Il23a*^*-/-*^ mice were infected intravenously with 2x10^5^ CFU *C*. *albicans*. Viability of kidney myeloid cells was assessed by flow cytometry at 48h post infection using 7-AAD and Annexin V reagents. (A) Representative FACS plots show the gating strategy for 7-AAD and Annexin V staining of neutrophils that were pre-gated without prior exclusion of dead cells. (A—C) Summary graphs show the percentage of 7-AAD^-^Annexin V^-^ and 7-AAD^+^Annexin V^+^ populations among total neutrophils, Ly6C^hi^ monocytes and Ly6C^lo^ myeloid cells. Neutrophils and Ly6C^hi^ monocytes were defined as shown in Fig 2C, while Ly6C^lo^ myeloid cells comprising all remaining myeloid cells were gated as CD45^+^CD11b^+^Ly6C^lo^Ly6G^-^. Bars are the mean + SD of each group with n = 4. Data are representative of three independent experiments. Statistics were calculated using unpaired Student’s t-Test. *p<0.05, **p<0.01, ***p<0.001.

The intriguing discrepancy that we observed in IL-23-deficient mice between the increased de novo generation and trafficking of myeloid cells and the strongly reduced numbers of these cells in the infected kidney prompted us to search for an alternative explanation for their rapid disappearance from the site of infection. We hypothesized that myeloid cells might have a survival defect in absence of IL-23. We therefore examined the viability of myeloid cells in the infected kidney at 48h post infection by means of 7-AAD and Annexin V staining and flow cytometry analysis *ex vivo*. In comparison to the situation in WT mice, we detected in *Il23a*^*-/-*^ mice a significantly reduced proportion of 7-AAD^-^Annexin V^-^ viable cells that was mirrored by an increased proportion of 7-AAD^+^Annexin V^+^ dead cells. This applied to all myeloid cell populations that were found to disappear in the infected kidney of IL-23-deficient mice at 48h post infection, including neutrophils **(Fig 4A)**, Ly6C^hi^ monocytes **(Fig 4B)** and the remaining Ly6C^lo^ monocytes, macrophages and DCs, collectively named Ly6C^lo^ myeloid cells **(Fig 4C)**. Dying cells are usually quickly removed from tissues in a process called efferocytosis [30]. This likely results in underestimation of the degree of myeloid cell death in the *C*. *albicans*-infected kidney of *Il23a*^*-/-*^ mice and the apparent lack of inflammatory infiltrates on histology sections **(Fig 2B)**. Importantly, there was no evidence for impaired cell viability prior to infection, as equal numbers of myeloid cells were present in BM, spleen and blood of naïve WT and *Il23a*^*-/-*^ mice at steady state **(S3A–S3C Fig)** and these populations did not differ in 7-AAD or Annexin V staining between the two genotypes **(S3D and S3E Fig)**. Likewise, we did not detect differences in neutrophil morphology or function between the two genotypes **(S4A–S4F Fig)**.

Next we were interested to understand the role of fungal virulence and of PAMPs in the IL-23-mediated protection from myeloid cell death in response to *C*. *albicans*. To this aim, we intravenously injected a yeast-locked mutant of *C*. *albicans* [31] into mice bearing or lacking a functional IL-23 pathway. Under these conditions, the infiltration of inflammatory cells into the kidney was much diminished in comparison to infection with the wild type strain SC5314 and no significant differences between the two mouse genotypes could be observed **(S5A and S5B Fig)**, indicating that the IL-23-dependent pro-survival mechanism was not relevant in the context of an attenuated inflammatory response to avirulent fungus. Next, we directly exposed bone marrow neutrophils in vitro to β-glucan or live and heat-killed *C*. *albicans*. Although these stimuli triggered significant degree of cell death in the cultured cells, there was no difference in viability between IL-23 pathway-sufficient and -deficient neutrophils **(S5C Fig)**, suggesting that direct contact with *C*. *albicans* or fungal PAMPs was not sufficient to reveal survival differences. Collectively, these results suggest that the loss of myeloid cells under IL-23-deficient conditions is a consequence of their impaired survival that becomes apparent only *in vivo* in the context of a strong inflammatory environment during the course of systemic infection with virulent *C*. *albicans*.

### IL-23-dependence of myeloid cell survival is not a general feature of systemic infections

**Fig 5:**
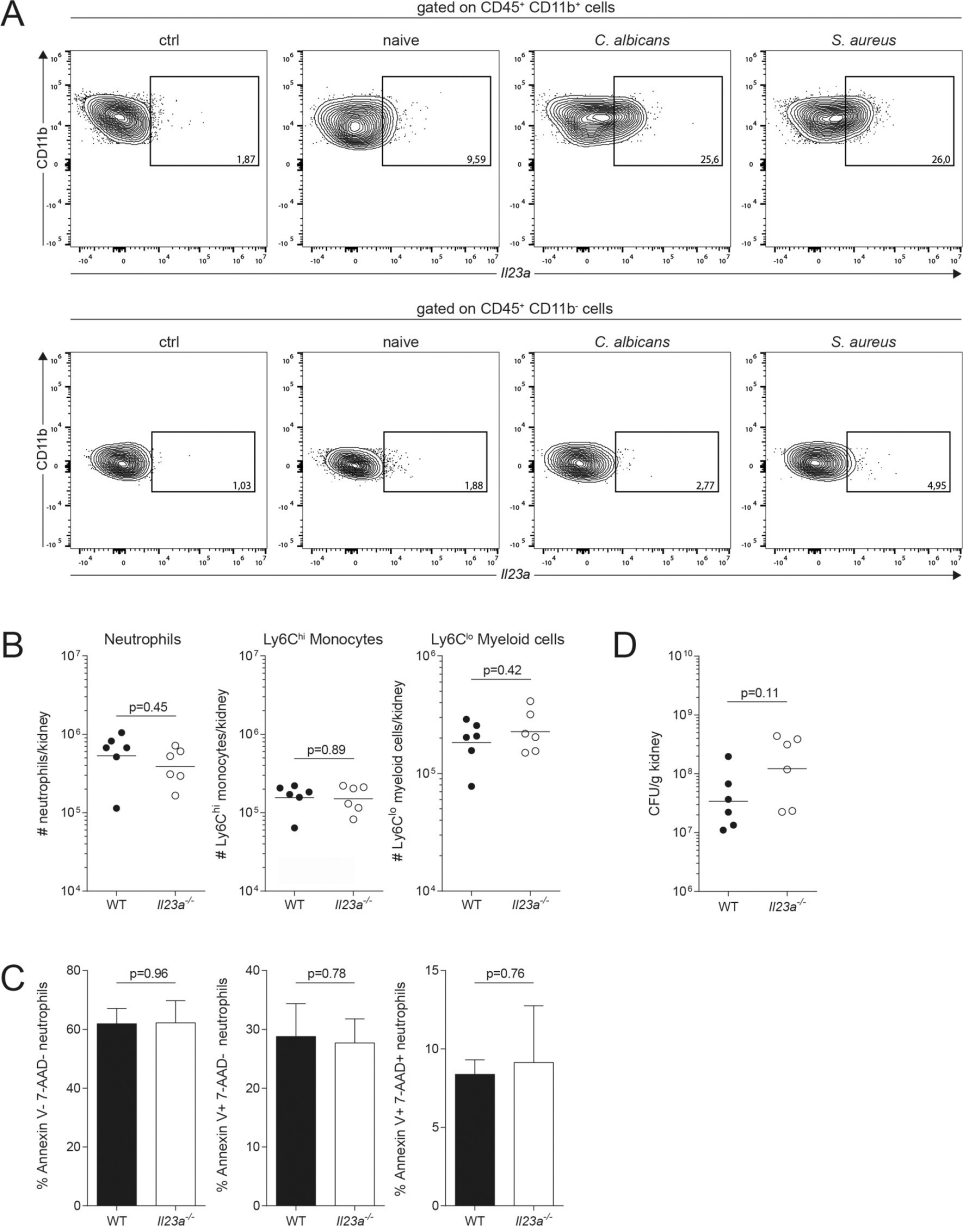
Systemic *S*. *aureus* infection does not impair myeloid cell viability in *Il23a*^*-/-*^ mice. (A) WT mice were infected intravenously with 2x10^5^ CFU *C*. *albicans*, 5x10^7^ CFU *S*. *aureus* or left uninfected (naive). Kidney cells were isolated at 24h post infection and *Il23a* mRNA expression was assessed by flow cytometry using the PrimeFlow RNA assay. Representative FACS plots show *Il23a* mRNA expression in CD11b^+^ and CD11b^-^ kidney leukocytes. A *C*. *albicans* infected sample without *Il23a* gene-specific target probe set served as a control (ctrl). (B—D) WT and *Il23a*^*-/-*^ mice were infected intravenously with 5x10^7^ CFU *S*. *aureus*. (B) Myeloid cell populations in the kidney were quantified by flow cytometry at 48h post infection. Neutrophils, Ly6C^hi^ monocytes and Ly6C^lo^ myeloid cells were defined as shown in Fig 2C. Summary graphs show the absolute numbers of each cell population per kidney. Each dot represents one animal and the mean of each group is indicated. (C) Viability of neutrophils was assessed by flow cytometry at 48h post infection using 7-AAD and Annexin V reagents as described in Fig 4A. Summary graphs show the percentage of 7-AAD^-^Annexin V^-^, 7-AAD^-^Annexin V^+^ and 7-AAD^+^Annexin V^+^ populations among total neutrophils. Bars are the mean + SD of each group with n = 3 and 4 respectively. (D) Kidney bacterial load was determined at 48h post infection. Each dot represents one animal. The mean of each group is indicated. Data in A and C are representative of two independent experiments. Graphs in B and D show pooled data from two independent experiments. Statistics were calculated using unpaired Student’s t-Test.

Next, we asked whether IL-23-dependent effects on myeloid cell viability were specific and restricted to *C*. *albicans* infection or a more general phenomenon. We therefore set out to compare myeloid cell viability in IL-23-sufficient and -deficient conditions in a different infection model. The bacterium *Staphylococcus aureus* is an opportunistic pathogen in humans that can cause invasive disease. It is well established that experimental systemic infection with *S*. *aureus* in mice leads to prominent kidney abscesses with comparable temporal dynamics as systemic candidiasis [32,33]. In addition, IL-23 has been reported to be induced in response to *S*. *aureus*, at least in a skin-infection setting [34]. Hence, we compared *Il23a* mRNA induction in the kidney of mice that were systemically infected with *S*. *aureus* or *C*. *albicans* for 24h. Using the PrimeFlow RNA assay, a novel flow cytometric assay that combines antibody staining and in situ hybridization of RNA transcripts for the detection of specific mRNA of interest in complex cell populations [35], we detected comparable levels of *Il23a* transcripts in response to both pathogens, which were ~2.5 times increased in CD11b^+^ myeloid cells from infected WT mice compared to naïve controls **(Fig 5A)**. In the following, we systemically infected WT and *Il23a*^*-/-*^ mice with *S*. *aureus* and analyzed kidney myeloid cells for their number and viability at 48h post infection. In sharp contrast to the situation during systemic candidiasis, we detected no reduction in any of the myeloid cell populations in *Il23a*^*-/-*^ mice compared to WT controls **(Fig 5B)** and the viability was comparable between both genotypes for all subsets **(Fig 5C)**. Accordingly, numbers of viable bacteria recovered from kidneys of *Il23a*^*-/-*^ and WT mice were comparable **(Fig 5D)**.

In the following, we were interested to know whether the IL-23-dependent effects on myeloid cell survival expanded to other fungal infections beyond *C*. *albicans*. To this aim, we made use of a recently developed experimental model of *Malassezia pachydermatis* skin infection [36]. Of note, we found that the viability of neutrophils was impaired in the skin of *M*. *pachydermatis*-infected mice in absence of a functional IL-23R pathway **(S6A Fig).** However, this did not result in a change of overall neutrophil numbers **(S6B Fig),** most likely due to the fact that experimental colonization of the murine skin with *Malassezia* is not accompanied with systemic inflammation. The supply of neutrophils from the circulation may thus be sufficient to replace the dying cells in the infected tissue of IL-23 signaling-deficient mice. Collectively, our data suggest that the dependency of myeloid cell survival on IL-23 is not a general phenomenon of systemic infection, but rather attributed to infections with fungi including both systemic and local infections.

### Myeloid cells die by apoptosis in absence of functional IL-23 pathway during systemic *C*. *albicans* infection

**Fig 6:**
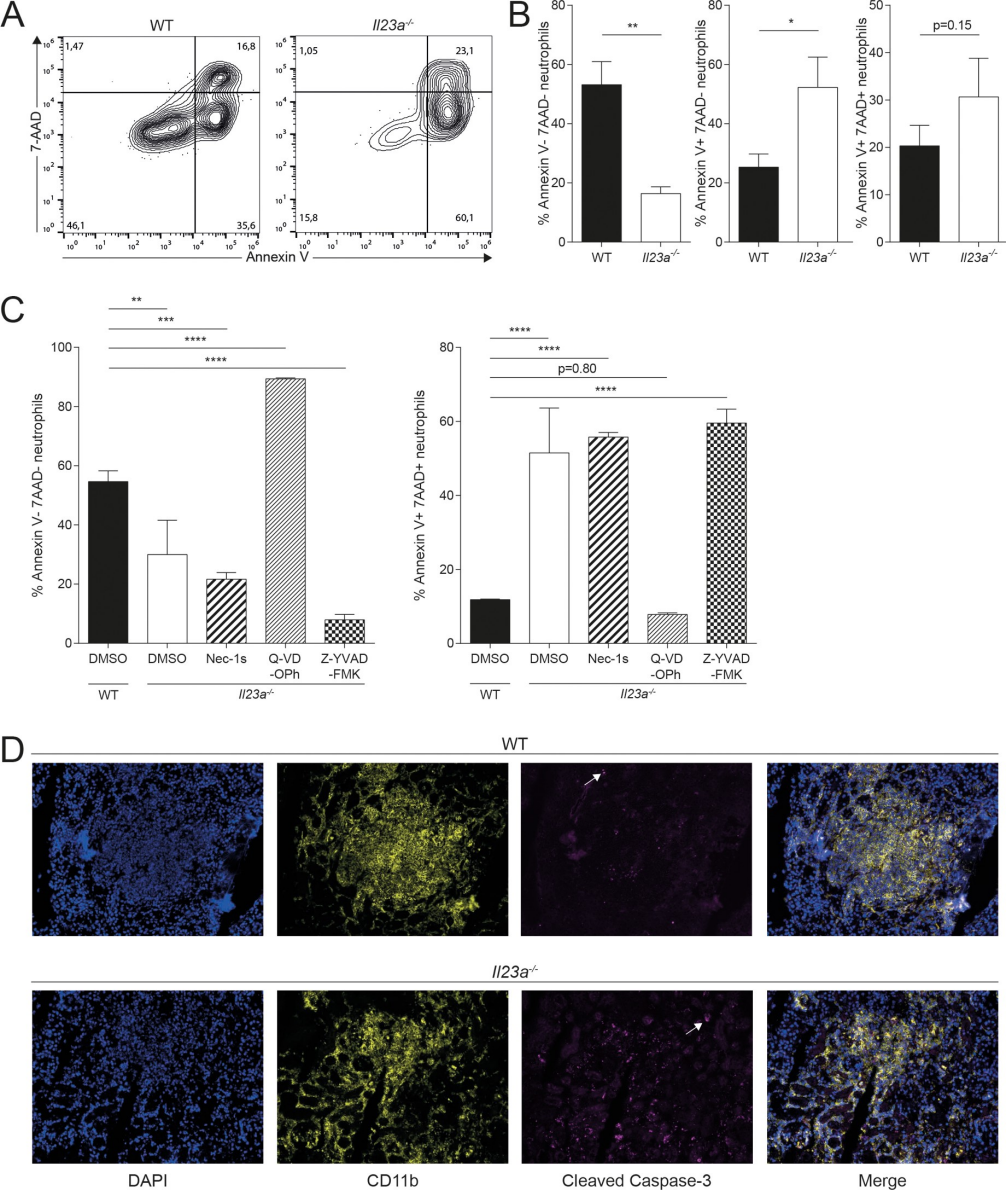
Impaired viability of neutrophils in infected *Il23a*^*-/-*^ mice is a consequence of increased apoptosis. WT and *Il23a*^*-/-*^ mice were infected intravenously with 2x10^5^ CFU *C*. *albicans*. Kidney neutrophils were isolated by density gradient centrifugation at 24h post infection and cultured in supplemented RPMI 1640 medium for 18h. Viability of neutrophils was assessed by flow cytometry using 7-AAD and Annexin V reagents. (A) Representative FACS plots are pre-gated on neutrophils as shown in Fig 2C without prior exclusion of dead cells. (B) Summary graphs showing the percentage of 7-AAD^-^Annexin V^-^, 7-AAD^-^Annexin V^+^ and 7-AAD^+^Annexin V^+^ populations among total neutrophils. Bars are the mean + SD of each group with n = 3. Data are representative of three independent experiments. (C) Neutrophils were purified from the BM of infected WT and *Il23a*^*-/-*^ mice at 24h post infection and cultured with the indicated cell death inhibitors or DMSO as a control in supplemented RPMI 1640 medium for 48h. The cell viability was then assessed as described in (A—B). Summary graphs show the percentage of 7-AAD^-^Annexin V^-^ and 7-AAD^+^Annexin V^+^ populations among total neutrophils. Bars are the mean + SD of each group with n = 3. Data are representative of two independent experiments. (D) WT and *Il23a*^*-/-*^ mice were infected intravenously with 2x10^5^ CFU *C*. *albicans*. Sagittal kidney sections were stained 48h post infection for DNA (DAPI; blue), CD11b^+^ cells (anti-CD11b; yellow) and apoptotic cells (anti-cleaved caspase-3, magenta). Arrows indicate cleaved caspase-3^+^ cells. Representative images are shown. Data are representative of two independent experiments. Statistics were calculated using unpaired Student’s t-Test (B) or one-way ANOVA (C) as appropriate. *p<0.05, **p<0.01, ***p<0.001.

Given the inverse correlation of the disappearance of myeloid cells and increased fungal burden in *Il23a*^*-/-*^ mice, we wondered if the loss of myeloid cell viability was a direct consequence of the massive accumulation of fungus in the kidney. To assess myeloid cell viability under conditions when fungal loads were comparable between *Il23a*^*-/-*^ and WT mice, we isolated kidney neutrophils at 24h post infection. At this time point, neutrophils were still equal in numbers in the kidney of both groups of mice **(S1A Fig)**. First, we confirmed that their viability was comparable between WT and *Il23a*^*-/-*^ mice and that their viability was not differentially affected by enrichment via density gradient centrifugation **(S7A Fig)**. We then followed the fate of the two populations of enriched neutrophils in culture in absence of the infectious context. Starting from equal viability after isolation, we observed a more rapid death of *Il23a*^*-/-*^ neutrophils after overnight incubation in comparison to their WT counterparts **(Fig 6A and 6B)**. This result paralleled our findings from direct analysis of viability at 48h post infection **(Fig 4A)**. They show that the increased cell death of neutrophils in absence of IL-23 is uncoupled from differences in fungal load between IL-23-sufficient and IL-23-deficient conditions.

Next, we were interested to understand the form of cell death by which neutrophils were dying in absence of IL-23. The increase in Annexin V^+^ staining suggested that neutrophils undergo apoptosis. However, the specificity of Annexin V for marking apoptotic cells has been questioned, as it has been reported to stain other forms of ongoing cell death [37]. We thus repeated the experiment described above with neutrophils isolated from WT and IL-23-deficient mice at 24h post infection and added different cell death inhibitors during *in vitro* culture. Consistent with our previous results, untreated *Il23a*^*-/-*^ neutrophils exhibited a rapid loss of viability while addition of Q-VD-OPh, a potent pan-caspase inhibitor [38], fully restored their viability **(Fig 6C)**. In contrast, Necrostatin-1s (Nec-1s), a specific RIPK1 inhibitor to prevent necroptosis [39] had no effect **(Fig 6C)**. Since pan-caspase inhibition does not allow to distinguish between caspase-3-dependent apoptosis and caspase-1-dependet pyroptosis, we employed the caspase-1-specific inhibitor Z-YVAD-FMK, which, in contrast to Q-VD-OPh, did not prevent but even accelerated cell death of neutrophils from IL-23-deficient mice **(Fig 6C)**. These results indicate that pyroptosis does not contribute to cell death in our system. Another form of cell death, which neutrophils can undergo in response to *C*. *albicans* hyphae, is NETosis [40]. Assessing NETosis by means of the cell impermeable dye Sytox Green [41] did however not reveal differences between WT or *Il23a*^*-/-*^ neutrophils that were isolated from infected mice at 24h post infection and put in contact with *C*. *albicans* hyphae *in vitro* **(S7B Fig)**, suggesting that NETosis does not contribute to the IL-23-dependent cell death phenotype.

We further assessed the mode of cell death in different myeloid populations of the kidney using the same assay as shown in Fig 6C. We found Q-VD-OPh to restore the viability of neutrophils and Ly6C^hi^ monocytes from *Il23a*^*-/-*^ mice to levels comparable to those of WT cells **(S7C Fig)**, confirming our previous results obtained with bone marrow neutrophils **(Fig 6C)**. Whether this also applies to kidney macrophages and DCs could not be assessed conclusively **(S7C Fig)**.

Finally, we assessed myeloid cell apoptosis *in situ* by immunofluorescence microscopy. In kidney sections of *Il23a*^*-/-*^ mice, we detected increased staining for the apoptosis marker cleaved caspase-3 within CD11b^+^ myeloid cell infiltrates compared to WT mice **(Fig 6D)**. Of note, inflammatory infiltrates were generally less dense in *Il23a*^*-/-*^ mice by means of CD11b and DAPI staining, indicating the reduced presence of myeloid cells. In summary, these results show that the rapid loss in viability of *Il23a*^*-/-*^ neutrophils is due to enhanced apoptosis during systemic candidiasis and they support the notion that an intact IL-23 pathway is required to prevent neutrophil apoptosis for optimal fungal control.

### IL-23-dependent prevention of myeloid cell death is independent of lymphoid cells and IL-17

IL-23 is well known as a key regulator of IL-17 immunity and the IL-23-IL-17 immune axis plays a pivotal role in host defense against *C*. *albicans* at mucosal surfaces [42–44]. IL-17 receptor-deficient mice are also highly susceptible to systemic candidiasis [13–15]. To assess whether the IL-17 pathway was involved in IL-23-dependent inhibition of myeloid cell apoptosis during systemic candidiasis, we infected *Il17ra*^*-/-*^ mice and analyzed the viability of myeloid cells 48h post infection **(S8A Fig)**. In sharp contrast to the situation in IL-23-deficient mice, IL-17RA deficiency did not affect the viability of kidney myeloid cells, with the exception of the Ly6C^lo^ myeloid subset, which exhibited a shift in the proportion of the 7-AAD- and Annexin V-stained populations. However, the absence of a difference in the overall number of viable Ly6C^lo^ myeloid cells in the kidney of *Il17ra*^*-/-*^ mice **(S8B Fig)** led us to conclude that the IL-17 pathway does not make a relevant contribution to the control of this myeloid cell subsets during systemic candidiasis. GM-CSF is another cytokine that can be regulated by IL-23 [16,17], as it has also been shown to be the case during systemic candidiasis [10] However, when analyzing *Csf2*^*-/-*^ mice, we could not gain evidence for the requirement of GM-CSF in promoting myeloid cell survival in the kidney of *C*. *albicans*-infected **(S8C Fig)**. If anything, GM-CSF seemed even to have an opposite effect and to prevent viability of Ly6C^hi^ monocytes. Together, our findings suggest an IL-17- and GM-CSF-independent function of IL-23 to be responsible for maintaining myeloid cell viability during systemic candidiasis.

The most well-known target cells of IL-23 are T cells including Th17 cells and γδ T cells as well as innate lymphoid cells (ILCs). However, by investigating *Rag*^*-/-*^*Il2rg*^*-/-*^ mice, which lack all cells of lymphoid origin, we ruled out a role of the lymphoid compartment in the IL-23-mediated effect as myeloid cell viability was not impaired in these mice when infected systemically with *C*. *albicans* **(S8D Fig)**.

**Fig 7:**
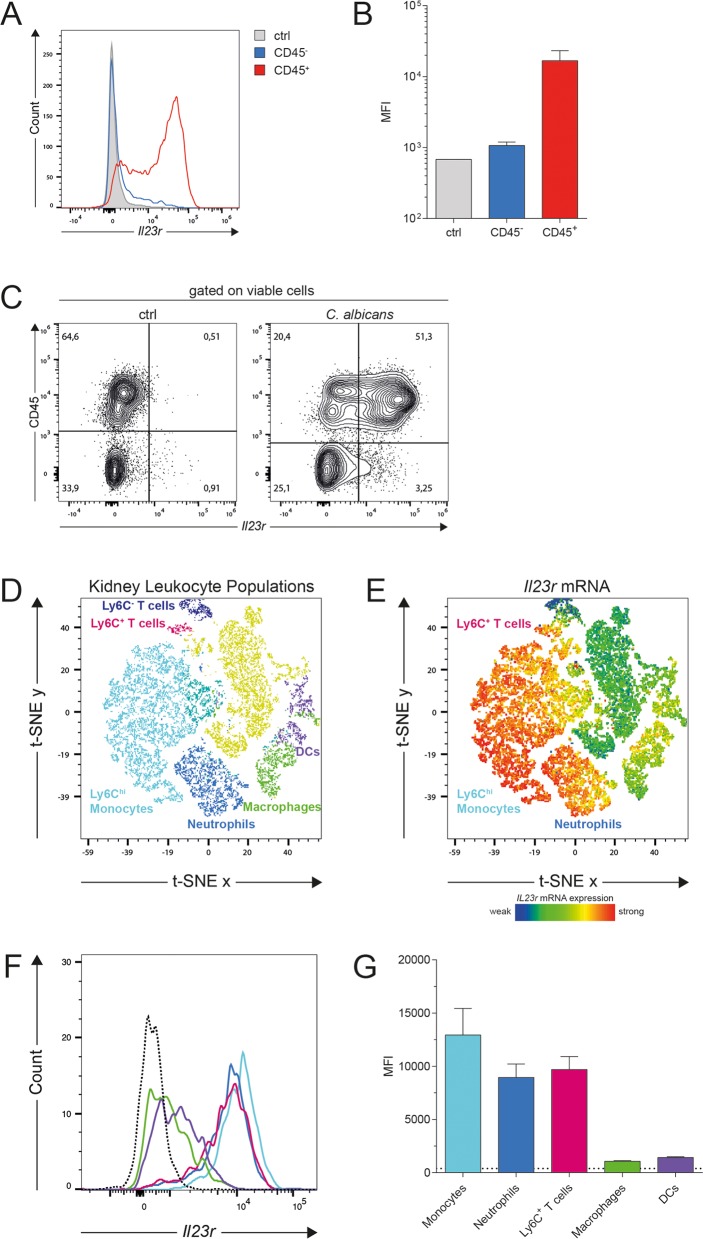
Ly6C^hi^ monocytes, neutrophils and Ly6C^+^ T cells express highest levels of *Il23r* transcripts in the infected kidney. WT mice were infected intravenously with 2x10^5^ CFU *C*. *albicans*. Kidney cells were isolated at 24h post infection and *Il23r* mRNA expression was assessed by flow cytometry using the PrimeFlow RNA assay. (A—B) Representative histogram (A) and summary graph (B) showing *Il23r* mRNA expression in CD45^-^ and CD45^+^ kidney cells and in a negative control population without *Il23r* gene-specific target probe set (ctrl). Bars are the mean + SD of n = 2 for CD45^-^ and CD45^+^ populations; n = 1 for the ctrl population. (C) Representative FACS plots show *Il23r* mRNA expression in CD45^-^ and CD45^+^ kidney cells. (D) t-SNE plot displaying CD45^+^ kidney leukocytes at 24h post infection analyzed by flow cytometry. Colored leukocyte clusters were identified using the gating strategy presented in Fig 2C and S1 Fig. (E—G) t-SNE plot (E), representative histograms (F) and summary graph (G) representing the median fluorescence intensity (MFI) of *Il23r* mRNA expression in the leukocyte clusters identified in (D). The dotted line indicates the MFI of pooled CD11b^+^ and CD3ε^+^ populations from a sample, in which the *Il23r* gene-specific target probe set was omitted. Bars are the mean + SD of n = 3. Data are representative of three independent experiments.

Given the unexpected lymphoid cell-independence of the IL-23-dependent effect on myeloid cell viability during systemic candidiasis, we set out to identify other cells that could potentially respond to IL-23 in the infected kidney. We first assessed IL-23 receptor expression in the hematopoietic and non-hematopoietic compartment in WT mice 24h post infection using the PrimeFlow RNA assay. We found that *Il23r* mRNA was predominantly expressed by CD45^+^ cells **(Fig 7A–7C)**. To further dissect IL-23 receptor expression on hematopoietic cells, we labeled cells with a panel of myeloid cell markers in combination with the PrimeFlow RNA assay and applied high-dimensional single-cell mapping [45]. In analogy to the gating strategy presented in **(Fig 2C)**, we identified populations of Ly6G^+^Ly6C^+^ neutrophils, Ly6C^hi^Ly6G^-^ monocytes, macrophages, DCs and two subsets of T cells **(Fig 7D)**. Interestingly, the highest levels of *Il23r* transcripts mapped to Ly6C^hi^ monocytes, neutrophils and a subset of Ly6C^+^ T cells, while macrophages and DCs expressed only very low levels of *Il23r* transcripts **(Fig 7E–7G)**. In line with our results, previous publications reported IL-23 receptor expression by cells of the myeloid lineages [20,25] Importantly, however, our data show that not all myeloid cell subsets, which are impaired in viability during systemic candidiasis in *Il23a*^*-/-*^ mice, express themselves high levels of *Il23r* transcripts **(Fig 7E–7G)**. This indicates that IL-23 unlikely acts in a cell-intrinsic manner to promote myeloid cell viability. To get further support for this possibility, we generated WT:*Il23r*^gfp/gfp^ mixed bone marrow chimeras and assessed the ratio of WT and *Il23r*^gfp/gfp^ neutrophils at 48h post infection in comparison to the situation prior to infection **(S9A and S9B Fig)**. If IL-23R signaling acts cell intrinsically, a shift towards a higher proportion of WT cells is expected due to a selective loss of the cellular compartment lacking the IL-23 receptor. However, our data show that the WT:*Il23r*^gfp/gfp^ ratio rather decreased than increased upon infection **(S9B Fig)** and that the viability of neutrophils and Ly6C^hi^ monocytes was comparable in both compartments **(S9C Fig)**. Of note, in case of the Ly6C^lo^ myeloid cell subset, we found the viability of the *Il23r*^gfp/gfp^ compartment to be slightly decreased, but the WT:*Il23r*^gfp/gfp^ ratio to be increased. At this stage, we are not able to explain the conflicting nature of this result, but given the fact that the WT:*Il23r*^gfp/gfp^ ratio did not increase, we believe that IL-23 is unlikely to act in a cell-intrinsic manner. Taken together, our data indicate that IL-23 promotes the viability of myeloid cells at the site of infection independently of the generic IL-23-IL-17 axis and well-characterized target cells of IL-23 within the lymphoid compartment, but potentially via Ly6C^hi^ monocytes and/or neutrophils. The fact that all myeloid cell subsets, and in particular neutrophils and Ly6C^hi^ monocytes are the primary source of IL-23 during systemic candidiasis **(S10 Fig)** and likewise express highest levels of *Il23r* transcripts **(Fig 7E–7G)** indicates that IL-23 possibly acts in partially autocrine but not cell-intrinsic manner within the myeloid compartment, along a novel and not yet fully characterized pathway.

## Discussion

IL-23 signaling is critically important for fungal control and host protection at the onset of systemic candidiasis. By harnessing the experimental model of systemic candidiasis in mice, we found that IL-23 regulates myeloid cell dynamics in the infected kidney. Absence of IL-23 led to a rapid loss of tissue-infiltrating neutrophils and monocytes as well as tissue-resident macrophages and dendritic cells from the infected kidney. Our results revealed that IL-23 was not required for maintaining the circulating pool of myeloid cells at steady state or for *de novo* myelopoiesis and recruitment of the newly generated cells to the site of infection. Instead, we provide evidence that IL-23 sustains the viability of myeloid cells in the infected kidney during systemic candidiasis. This effect was independent of canonical IL-23 target cells such as those of the lymphoid lineage. Instead, we found that the IL-23 receptor is prominently expressed by neutrophils and Ly6C^hi^ monocytes, suggesting that these cells are responsible for the observed IL-23-mediated effects in a non-cell-intrinsic manner. Collectively, our data reveal a novel function of IL-23 that ensures myeloid cell survival during systemic candidiasis and thereby plays a central role in coordinating the myeloid compartment against *C*. *albicans* for optimal host protection.

Cytokines are instrumental for orchestrating the timely recruitment and appropriate activation of myeloid effector cells at the site of infection. Tight regulation of this system is a prerequisite for protective antifungal defense as perturbations of the fine line between host protection and immunopathology can have detrimental consequences for the host. The spatiotemporal accumulation of diverse myeloid cell subsets in the infected kidney has been documented [27], and key innate immune populations required for host protection from systemic candidiasis and trafficking of these populations from their reservoir to the site of infection have been identified [6–8,10]. Novel mechanisms of fungal killing and the regulation of antifungal effector mechanisms by myeloid cell subsets have been proposed [40,46]. Yet, little is known about how myeloid effector cells are maintained at the site of infection during the presence of the pathogen and how they are removed after fungal clearance to restore homeostasis. Here, we propose a novel function of IL-23 as a key regulator of myeloid cell dynamics in systemic candidiasis.

IL-23 is induced in response to fungal recognition by C-type lectin receptors upon tissue invasion of *C*. *albicans* [9,10]. Our data show that IL-23 is key for preventing myeloid cell apoptosis in the infected kidney. The rapid loss of myeloid cells shortly after the onset of infection if a functional IL-23 pathway is missing suggests that the dying/dead myeloid cells are rapidly removed from the site of infection. The extent of cell death that we observed in *Il23a*^*-/-*^ mice is thus likely underestimated. Together, our results suggest that IL-23 ensures the presence of myeloid cells in the infected kidney as long as the infection persists. In a broader sense, IL-23 could therefore be regarded as a rheostat of myeloid cell viability, *i*.*e*. myeloid cell presence at the site of infection, to minimize variations in myeloid cell numbers that may otherwise compromise host protection or promote immunopathology during systemic *C*. *albicans* infection. These results are complementary to, but fully independent of a previous report that proposed that IL-23 contributes to antifungal defense by promoting neutrophil activity via an NK cell- and GM-CSF-dependent pathway [10].

IL-23 is involved in immunity to a variety of infectious diseases and it also promotes inflammatory disorders. However, to our knowledge, IL-23 has not been linked to cell viability so far. To explore whether IL-23 was generally required for maintaining myeloid cell viability during infectious conditions, we employed models of systemic infection with *S*. *aureus* and of local infection with *M*. *pachydermatis*. Although similar levels of IL-23 were induced in response to *S*. *aureus* as with *C*. *albicans*, we could not recapitulate the phenotype of decreased myeloid cell survival that we observed in IL-23- and IL-23R-deficient mice during systemic candidiasis, indicating again that the role of IL-23 in preventing myeloid cell death was not conserved during systemic infections. In contrast, the IL-23 pathway seemed to have a similar role in promoting myeloid cell survival during skin colonization with *Malassezia* than during systemic candidiasis. Interestingly, systemic infection of *Il23a*^*-/-*^ mice with the fungus *Cryptococcus neoformans*, which has a strong tropism for the CNS, was reported to lead to reduced density of inflammatory infiltrates in the CNS [47]. This may possibly be explained by a mechanism reminiscent of the one described in this paper. Assessing the role of IL-23 in additional infectious and inflammatory disease models in the future will help to clarify to what extent IL-23-mediated maintenance of myeloid cell viability might be limited to fungal infections and what might be the fungus-specific components of this effect.

The best known function of IL-23 is to promote IL-17 production by T cells and ILCs. It is thus conceivable that IL-23 could act via induction of innate IL-17 during systemic *C*. *albicans* infection, as it has been shown to be the case in oral [42,43] and dermal [48] *C*. *albicans* infection models. However, IL-17 is only weakly induced at the onset of systemic candidiasis [14]. Although the requirement for the IL-17 pathway in protection from systemic *C*. *albicans* infection was documented already 15 years ago [13], only a few studies addressed the mode of action of IL-17 during infection [14,15].

Given the capacity of IL-17 to regulate neutrophil recruitment into tissues, it appeared obvious at first to assume that the strong reduction in myeloid cell numbers in the kidney of IL-23-deficient mice would be the result of dysregulated IL-17 expression. This was also supported by Huang et al., who observed reduced accumulation of neutrophils in the kidney of *C*. *albicans*-infected IL-17 receptor deficient mice [13]. However, several arguments speak against this possibility. First, the role of IL-17 in regulating the recruitment of neutrophils is controversial, as several reports showed that neutrophil recruitment is not always IL-17-dependent [49,50]. Accordingly, our own results showed that during systemic candidiasis IL-17 receptor-deficient animals exhibited normal numbers of neutrophils in the kidney. Second, the observed dynamics of myeloid cell numbers at the site of infection do not support a recruitment defect in IL-23-deficient mice as we found comparable numbers of myeloid cells early during infection and even detected an increased supply of newly generated neutrophils in the BM and at the site of infection. Third, we considered that IL-23-mediated effects on myeloid cells might take place during steady state prior to infection. This hypothesis was stimulated by a previous report on an IL-23-mediated feedback loop affecting neutrophil homeostasis during steady state conditions [51]. However, in our colony of mice, IL-23-deficiency did not affect neutrophil numbers or activity in the BM or in the circulation under steady-state conditions. We thus assume redundancy in the regulation of homeostatic granulopoiesis, which, in our hands, did not depend on IL-23. Instead, we found IL-23 to regulate myeloid cell viability after the onset of systemic infection with *C*. *albicans*.

Our results fit in a growing list of examples of additional roles for IL-23. A recent report suggested an IL-17-independent IL-23-dependent protective pathway in the context of experimental oropharyngeal candidiasis, which was linked to IL-36 [52]. Two other pivotal studies showed that IL-23 instructs IL-22 production, which in turn promotes dermal inflammation and acanthosis [19] and that IL-23 can drive GM-CSF production, which is essential for the induction of EAE [16,17]. In both cases, IL-23 acts directly on T cells. These studies thus revealed an extended repertoire of T cell-derived IL-23-dependent cytokines. We ruled out the possibility that the effect of IL-23 on myeloid cell viability was mediated via T cells or other lymphoid cells. Thus, in our setting, IL-23 seems to act in a fundamentally different way from what was reported before. The literature on T-cell independent effects of IL-23 is scarce. A few reports suggest however that IL-23 can act on myeloid cells, in particular on neutrophils. It was suggested that IL-23 regulates cytokine production by neutrophils during experimental mold infections [20,21]. Subsequent studies confirmed the requirement of IL-23 for IL-17 production by neutrophils [22,24]. Another study suggested that IL-23 induces osteoclast differentiation in the mouse macrophage cell line RAW264.7 via an IL-17-independent mechanism [23]. In line with an increasing number of examples, the work presented in this study suggests that IL-23 might act directly on myeloid cells.

Although myeloid cells possibly respond directly to IL-23, experiments with mixed chimeras with BM from WT and IL-23R-deficient mice indicated that IL-23 does not regulate myeloid cell viability in a cell-intrinsic manner. Consistent with this, we detected IL-23R transcripts in some but not all of the myeloid cell subsets that succumbed to death during systemic candidiasis in absence of IL-23. In particular, we found high levels of *Il23r* transcripts in neutrophils and Ly6C^hi^ monocytes, but not in macrophages and DCs, which were however also affected by rapid cell death in absence of a functional IL-23 pathway. Of note, we could not confirm *Il23r* expression in NK cells from *C*. *albicans*-infected kidneys reported previously [10]. Further investigation revealed that the antibody previously used for staining IL23R on NK cells [10] is not fully specific. Combining the results from the previous publication [10] with those of our current study, we suppose that the originally described effect of IL-23 on NK cells is likely to be indirect and mediated via myeloid cells, which express the highest levels of IL-23R in the *C*. *albicans*-infected kidney. We propose a model in which IL-23R-expressing myeloid cells act as coordinators of IL-23-dependent innate immunity during systemic candidiasis by inducing GM-CSF production by NK cell and by providing survival signals to all myeloid cell subset in the infected kidney to secure their survival for enhanced fungal control.

Putative survival signals for myeloid cells remain to be identified. GM-CSF is a promising candidate that acts on diverse myeloid cell types [53] to prolong their survival by inhibition of apoptosis [54]. However, our results indicate that GM-CSF is dispensable for IL-23-induced myeloid cell survival during systemic candidiasis. G-CSF, M-CSF, TNF and IL-3 can also promote the viability of myeloid cells [55–57] and may therefore represent interesting candidates to be investigated in the future. Conversely, it is conceivable that IL-23 might negatively regulate factors that promote apoptosis of myeloid cells. In such a scenario, the lack of IL-23 during infection would lead to increased production of pro-apoptotic factors, thus resulting in increased apoptosis of myeloid cells. In support of such a scenario, we found that the IL-23-dependent phenotype was observed only with high-virulent fungus and preferentially at high infection doses, which are associated with a high degree of inflammation. This indicates that strong inflammation is required for the IL-23-dependent protection from myeloid cell apoptosis. This notion would fit a scenario in which the massive inflammation during systemic *C*. *albicans* infection in IL-23-deficient mice would lead to overt production of pro-apoptotic factors promoting myeloid cell death and removal. It is also conceivable that a combination of several pro- and/or anti-apoptotic factors, including the aforementioned ones and others, would account for myeloid-specific cell death. Further research is warranted to dissect the regulatory program that underlies the IL-23-mediated survival of myeloid cells during systemic *C*. *albicans* infection that we identified in this study.

## Material and methods

### Reagents

All chemicals, reagents, antibodies, mouse strains, fungal and bacterial strains, instruments and software used in this study are listed in **S1 Table**.

### Ethics statement

All mouse experiments in this study were conducted in strict accordance with the guidelines of the Swiss Animals Protection Law and were performed under the protocols approved by the Veterinary Office of the Canton Zurich, Switzerland (license number ZH201/2012, ZH183/2015 and ZH167/2018). All efforts were made to minimize suffering and ensure the highest ethical and humane standards. Animals were euthanized by CO_2_.

### Animals

WT C57BL/6J mice were purchased from Janvier Elevage. *Il23a*^*-/-*^ mice [58], *Il23r*^gfp/gfp^ (which represent IL-23R-deficient mice if the GFP-knock-in allele is bred to homozygosity) [25], *Il17ra*^*-/-*^ mice [50] *Rag2*^*-/-*^*Il2rg*^*-/-*^ mice [59,60], *Csf2*^*-/-*^ mice [61] were maintained and bred at the Institute of Laboratory Animals Science of the University of Zurich, Switzerland. *Il17ra*^*-/-*^ mice were obtained via an MTA between the University of Zurich and Amgen (Thousand Oaks, CA). All mice were on the C57BL/6 background, kept in specific pathogen-free conditions and used at 6–12 weeks of age in age-matched groups. Female, as well as male mice, were used for experiments.

For generation of mixed bone marrow (BM) chimeras, 6- to 8-week-old WT (CD45.2^+^) recipient mice were irradiated 2x with a dose of 5.5 Gy at an interval of 12h. Next, femurs and tibias from 6- to 8-week-old WT (CD45.1^+^) and *Il23r*^gfp/gfp^ (CD45.2^+^) donor mice were removed, BM was flushed with PBS under sterile conditions and mixed at equal ratios (50:50). Recipient mice were reconstituted by lateral tail-vein injection of mixed donor BM 18h after the second irradiation. The first 2 weeks of reconstitution, mice were given Borgal (MSD Animal Health GmbH) in the drinking water. Mixed BM chimeras were used for experiments 10 weeks after reconstitution.

### *C*. *albicans* culture conditions and infection

Unless stated otherwise, the *C*. *albicans* laboratory strain SC5314 [62] was used for all experiments. *C*. *albicans* was grown in yeast peptone D-glucose (YPD) medium at 30°C and 180 rpm for 15–18h. Mice were infected via the lateral tail vein with 2 x 10^5^ *C*. *albicans* yeast cells (*Il23a*^*-/-*^ mice) or 3 x 10^5^ *C*. *albicans* (*Il23r*^gfp/gfp^ mice). Mice were monitored for morbidity throughout the course of all experiments. For determination of fungal burden, organs of euthanized animals were removed, homogenized in sterile 0.05% NP40 in H_2_O for 3 min at 25 Hz using a Tissue Lyzer (Qiagen) and serial dilutions were plated on YPD agar containing 100 **μ**g ml^−1^ ampicillin. For induction of hyphae, 5 × 10^3^ cfu *C. albicans* yeast cells were incubated in Hanks’ Balanced Salt Solution (Life Technologies) containing CaCl_2_ and MgCl_2_ and supplemented with 5% FCS for 3h at 37°C in a humidified atmosphere containing 5% CO2.

### *S*. *aureus* culture conditions and infection

The *S*. *aureus* laboratory strain Newman [63] was grown in tryptic soy broth medium at 37°C and 220 rpm for 15–18h. Subsequently, bacteria were diluted 1:10 in fresh medium and grown for an additional 2h to obtain bacteria in exponential growth phase. Mice were then infected via the lateral tail vein with 5 x 10^7^ bacteria. Mice were monitored for morbidity throughout the course of all experiments. For determination of bacterial load, organs of euthanized animals were removed, homogenized in sterile 0.05% NP40 in H_2_O for 3 min at 25 Hz using a Tissue Lyzer (Qiagen) and serial dilutions were plated on tryptic soy broth agar.

### *M*. *pachydermatis* culture conditions and infection

The *M. pachydermatis* strain ATCC 14522 [64] was grown in liquid modified Dixon medium at 30°C and 180 rpm for 3–4 days. Cells were washed in PBS and suspended in olive oil (native olive oil extra, SPAR) at a density of 20 OD_A600_/ml. The dorsal ear skin was disrupted by mild tape stripping (Transpore Hypoallergenic, 3M; 5 rounds per ear) and100 μl suspension (corresponding to 2 OD_A600_) of yeast cells was applied topically onto the dorsal ear skin while mice were anaesthetized.

### Histology

Mice were euthanized and kidneys were removed, fixed in 4% PBS-buffered paraformaldehyde overnight and embedded in paraffin. For histology, tissue sections (3–5 μm) were mounted on glass slides, stained with Periodic-Acid Schiff (PAS) reagent, counterstained with hematoxylin and mounted with Pertex (Biosystem, Switzerland) according to standard protocols. Images were acquired with a digital slide scanner (NanoZoomer-XR C12000; Hamamatsu) and analyzed with the NDP.view2 software (Hamamatsu).

### Immunofluorescence

For immunofluorescence staining, mice were euthanized and perfused with PBS. Kidneys were removed and embedded in Tissue-TEK O.C.T compound (VWR International GmbH, Switzerland), snap frozen in liquid nitrogen and stored at -20°C. Sagittal cryosections (6 μm) were cut and mounted to super frost glass slides (Thermo Scientific). The specimens were allowed to dry at room temperature and subsequently fixed with methanol at -20°C for 15 minutes. Sections were then permeabilized with 0.1% Triton X-100 for 5 min and blocked with 5% goat serum for 2h at room temperature, before staining with rat anti-CD11b (M1/70, Biolegend) and anti-cleaved Caspase-3 (Asp175) (5A1E, Cell Signaling Technology). Secondary antibodies were goat anti-rat Cy3 (Jackson ImmunoResearch) and goat anti-rabbit-AF647 (Jackson ImmunoResearch). DNA was stained with 4′,6′-Diamidino-2-phenylindole dihydrochloride (DAPI, Sigma-Aldrich). The stained specimens were washed with distilled H_2_O, mounted with Mowiol and stored at 4°C until analysis. All images were acquired with a Vectra 3.0 Automated Quantitative Pathology Imaging System (PerkinElmer) and analyzed with Phenochart (PerkinElmer) and inForm (PerkinElmer) software.

### Analysis of kidney functional markers and G-CSF in serum

Blood was collected from infected mice by cardiac puncture and serum was obtained by centrifugation of the clotted blood for 90 seconds at 15’000 g and stored at -20°C until analysis. Blood urea nitrogen (BUN) and creatinine were measured colorimetrically using the QuantiChrom Urea and QuantiChrom Creatinine assay kits (BioAssay Systems) according to the manufacturer’s instructions. G-CSF protein was determined by sandwich ELISA using purified anti-G-CSF (clone 67604, R&D Systems) for coating and biotinylated polyclonal rabbit anti-G-CSF (Peprotech) for detection according to standard protocols.

### Preparation of kidney, brain, bone marrow and blood cells for flow cytometric analysis

Mice were euthanized and perfused with PBS for kidney leukocyte quantification. Kidneys and brains were cut into fine pieces, digested with DNase I (200 μg/ml, Roche) and Collagenase I (240 mg/ml, Invitrogen) in RPMI 1640 at 37°C for 20 minutes and filtered through a 70 μm strainer. BM was flushed from femurs and tibiae and passed through a 70 μm strainer using ice-cold PBS supplemented with 1% FCS and 2 mM EDTA. Blood was collected by cardiac puncture. The erythrocytes in single cell suspensions from kidneys, BM and blood were removed by lysis in erythrocytes lysis buffer (0.3M NH_4_Cl, 28 μM NaHCO_3_, 125 μM EDTA) and erythrocyte-free single cell suspensions were then further processed.

### Preparation of ear skin cells for flow cytometric analysis

For digestion of total ear skin, ears of euthanized mice were removed, cut into small pieces and transferred into Hank’s medium (Ca2^+^- and Mg2^+^-free, Life Technology), supplemented with Liberase (0.15 mg/ml, Roche) and DNase I (0.12 mg/ml, Sigma-Aldrich) and incubated for 1 hour at 37 C. The cell suspension was filtered through a 70 μm cell strainer (Falcon) and rinsed with PBS supplemented with 5 mM ETDA (Life Technologies) and 1% FCS to be further processed.

### Enrichment of kidney leukocytes

Kidneys of euthanized mice were cut into fine pieces and digested with DNase I (200 μg/ml, Roche) and Collagenase I (240 mg/ml, Invitrogen) in RPMI 1640 at 37°C for 20 minutes. Single cell suspensions were passed through a 70 μm strainer using ice-cold PBS supplemented with 1% FCS and 2 mM EDTA. Leukocytes were enriched over a 40%-70% Percoll (Sigma Aldrich) gradient by density centrifugation at 650 g for 30 minutes without brake.

### Isolation of bone marrow neutrophils

BM was flushed from femurs and tibiae of euthanized mice and passed through a 70 μm strainer using ice-cold PBS supplemented with 1% FCS and 2mM EDTA. Neutrophils were isolated over a Histopaque-1077 and Histopaque-1119 (Sigma Aldrich) gradient by density centrifugation at 700 g for 30 minutes without brake. The polymorphonuclear cell fraction was collected and erythrocytes were lysed using erythrocytes lysis buffer (0.3M NH_4_Cl, 28 μM NaHCO_3_, 125 μM EDTA). Neutrophil preparations were consistently >80% pure.

### Neutrophil cell culture

Neutrophils were enriched from BM or kidneys by density gradient centrifugation as described above, resuspended in RPMI 1640 medium supplemented with 2 mM glutamine, 1% non-essential amino acids, 1% sodium pyruvate, 1% penicillin/streptomycin (all from Life Technologies), 5% FCS and incubated for the indicated time at 37°C in a humidified atmosphere containing 5% CO_2_. For inhibition of cell death, cells were treated with 50μM Nec-1s (Merck Millipore), 50μM Q-VD-OPh hydrate (Sigma-Aldrich) or 50μM Z-YVAD-FMK (Abcam) dissolved in DMSO. For neutrophil stimulation *in vitro*, enriched BM neutrophils were added to to 5 × 10^3^ preformed viable or heat-killed *C*. *albicans* hyphae or were stimulated with 100μg/ml curdlan in supplemented RPMI 1640 medium at 37°C in a humidified atmosphere containing 5% CO_2_ for 24h.

### Flow cytometry

Single cell suspensions of kidneys, BM and blood cells were stained in ice-cold PBS supplemented with 1% FCS, 5 mM EDTA and 0.02% NaN_3_. LIVE/DEAD Near-IR stain (Life Technologies) was used for exclusion of dead cells. The antibodies used for surface staining are listed in the **S1 Table**. For intracellular cytokine staining, cells were fixed and permeabilized using BD Cytofix/Cytoperm reagent (BD Bioscience) and subsequently incubated in Perm/Wash buffer (BD Bioscience). All extracellular and intracellular staining steps were carried out on ice. Cells were analyzed on a FACS Gallios (Beckman Coulter), Sony SP6800 Spectral Analyzer (Sony Biotechnology) or FACS LSR II Fortessa (BD Biosciences) and the data were analyzed with FlowJo software (Tristar). The gating of the flow cytometric data was performed according to the guidelines for the use of flow cytometry and cell sorting in immunological studies [65], including pre-gating on viable and single cells for analysis. Absolute cell numbers were calculated based on a defined number of counting beads (BD Bioscience, Calibrite Beads), which were added to the samples before flow cytometric acquisition. For analysis of myeloid cell viability, cells were first stained with antibodies for surface markers and then resuspended in Annexin V binding buffer (Biolegend) containing Annexin V reagent (Biolegend) and 7-AAD viability staining solution (Biolegend). After 15 min of incubation at room temperature in the dark, cells were analyzed on a FACS Gallios (Beckman Coulter) and the data were analyzed with FlowJo software (Tristar). Cells were pre-gated on single cells for analysis without prior exclusion of dead cells.

### BrdU incorporation assay

12h prior to euthanasia, mice were treated with 5-Bromo-2´-Deoxyuridine (BrdU; BD Pharmingen) by intraperitoneal injection (2mg) once and then *ad libitum* in drinking water (0.8mg/ml) until euthanasia. Kidney and BM cells were prepared as described above and BrdU incorporation was measured by flow cytometry using the APC BrdU Flow Kit (BD Pharmingen) according to manufacturer's instructions.

### PrimeFlow RNA assay

*In situ* RNA hybridization with enriched kidney leukocytes (see above) was performed using the PrimeFlow RNA Assay kit (Thermo Fisher Scientific) according to the manufacturer’s instructions. In brief, single cell suspensions of kidney leukocytes were surface-stained in ice-cold PBS supplemented with 1% FCS, 5 mM EDTA and 0.02% NaN_3_, subsequently fixed and permeabilized. RNA targets were stained by incubation with gene-specific target probes, pre-amplification, amplification and hybridization with fluorescently labeled probes. Cells were acquired on a FACS LSR II Fortessa (BD Biosciences) or a Sony SP6800 Spectral Analyzer (Sony Biotechnology) and the data were analyzed with FlowJo software (Tristar) as described above, including pre-gating on viable and single cells for analysis.

### ROS assay

2 x 10^5^ BM neutrophils were added to each well of a 96-well microplate containing 10^5^ *C*. *albicans* yeast cells or HBSS control. Total ROS production was measured by adding luminol (100 μM, Sigma-Aldrich). Chemiluminescence was measured on an Infinite 200 plate reader (Tecan) every 2.5 minutes over a total period of 2.5h starting immediately after addition of luminol.

### Neutrophils killing assay

1 x 10^5^ or 2 x 10^4^ neutrophils in HBSS were added to each well of a 96-well microplate containing 5 x 10^3^ *C*. *albicans* preformed hyphae and incubated for 4h at 37°C in a humidified atmosphere containing 5% CO_2_. The neutrophils were then lysed with water supplemented with 0.02% Triton-X-100 and the metabolic activity of *C*. *albicans* was assessed with the WST-1 cell proliferation reagent (Roche) according to the manufacturer’s instructions. After addition of WST-1 reagent, cells were incubated for 1h at 37°C and absorbance was read at 450 nm with a wavelength correction set at 630 nm. A standard curve was generated with serial dilutions of hyphal cells (without neutrophils). Killing activity was calculated as percent of viable (metabolically active) fungi in the presence of neutrophils compared to the conditions without neutrophils.

### Quantification of NET release from activated neutrophils

10^5^ freshly isolated BM neutrophils were added to 96-well tissue culture plates containing 5 × 10^3^ preformed *C*. *albicans* hyphae in HBSS containing CaCl_2_ and MgCl_2_ and incubated for 2.5 h at 37°C. Sytox Green (160 nM, Thermo Fisher) was added to the cells for detection of extracellular DNA. Unstimulated neutrophils were used as controls. The plates were analyzed on an Infinite 200 plate reader (Tecan) with excitation at 485 nm and emission at 535 nm. The fluorescence of stimulated cells was calculated by subtracting the baseline fluorescence of unstimulated cells and is expressed in arbitrary units.

### Statistics

Statistical significance was determined by unpaired Student’s t-test with Welch’s correction and one- or two-way ANOVA with Dunnet’s or Tukey’s multiple comparison test, as appropriate, using GraphPad Prism software. Data displayed on a logarithmic scale were log-transformed before statistical analysis. Outlier calculation was performed using the ROUT method. Significance is indicated as follows: *p< 0.05; **p<0.01; ***p<0.001; ****p<0.0001.

### Raw data

All raw data and metadata linked to this study are made publicly available on Zenodo: https://doi.org/10.5281/zenodo.3492252

## Supporting information

S1 Fig Numbers of myeloid cells at 24h post infection and lymphoid cells at 48h post infection are comparable in the kidney of infected WT and *Il23a*^*-/-*^ mice, but numbers of neutrophils are reduced in the brain of *C*. *albicans*-infected *Il23a*^*-/-*^ mice at 48h post infection. WT and *Il23a*^*-/-*^ mice were infected intravenously with 2x10^5^ CFU *C*. *albicans*. (A) Neutrophils and Ly6C^hi^ monocytes were quantified by flow cytometry at 24h post infection. (B-C) Lymphoid cell populations in the kidney were quantified by flow cytometry 48h post infection. Representative FACS plots in (B) show the gating strategy for kidney lymphoid cells. TCRβ^+^ T cells (P5), TCRγδ^+^ T cells (P6), ILCs (P7) and NK cells (P8) were defined as indicated. Summary graphs in (C) show the absolute numbers of each cell population per kidney. (D) Myeloid cell populations in the brain were quantified by flow cytometry at 48h post infection. Neutrophils and Ly6C^hi^ monocytes were defined as shown in Fig 2C. Ly6C^lo^ myeloid cells in the brain correspond by the large majority to microglia, which are defined by their low expression of CD45. (E) Representative FACS plot showing CD11b and CD45 expression in neutrophils, Ly6C^hi^ monocytes and microglia, gated as described in (D). In A, C and D, each dot represents one animal and the mean of each group is indicated. Data are pooled from two independent experiments. Statistics were calculated using unpaired Student’s t-Test. *p<0.05.
(TIF)

S2 Fig Emergency granulopoiesis and neutrophil supply is comparable between WT and *Il23a*^*-/-*^ mice at 24h post infection. WT and *Il23a*^*-/-*^ mice were infected intravenously with 2x10^5^ CFU *C*. *albicans*. Mice were treated with BrdU starting from 12h post infection and neutrophil proliferation was assessed 24h post infection. (A) Schematic representation of experimental design. (B) Summary graphs show percentage of BrdU^+^ cells within the total population of BM neutrophils (left) or kidney neutrophils (right). Neutrophils were defined as shown in Fig 2C. Bars are the mean + SD of each group with n = 3. Statistics were calculated using unpaired Student’s t-Test.
(TIF)

S3 Fig No difference in myeloid cell numbers and viability between naïve WT and *Il23a*^-/-^ mice. (A-C) Myeloid cell populations in (A) bone marrow, (B) blood and (C) spleen of naive WT and *Il23a*^*-/-*^ mice were quantified by flow cytometry. Neutrophils, Ly6C^hi^ monocytes and Ly6C^lo^ myeloid cells were defined as shown in Fig 2C. Summary graphs show the absolute numbers of each cell population per kidney. Each dot represents one animal and the mean of each group is indicated. (D, E) Neutrophils were purified from the bone marrow of naïve WT and *Il23a*^*-/-*^ mice and cultured in supplemented RPMI 1640 medium for 18h. Viability of neutrophils was assessed by flow cytometry using 7-AAD and Annexin V reagents. (D) Representative FACS plots of neutrophils that were pre-gated on neutrophils as shown in Fig 2C without prior exclusion of dead cells. (E) Summary graphs show the percentage of 7-AAD^-^Annexin V^-^ and 7-AAD^+^Annexin V^+^ populations among total neutrophils. Bars are the mean + SD of each group with n = 3. Statistics were calculated using unpaired Student’s t-Test.
(TIF)

S4 Fig Neutrophil function and morphology is comparable between WT and *Il23a*^*-/-*^ mice. (A) Reactive oxygen species (ROS) production by WT and *Il23a*^-/-^ bone marrow neutrophils in response to *C*. *albicans* yeast was detected by chemiluminescence using luminol reagent. Curves are the mean + SD of each group with n = 3. (B—C) Representative histogram in (B) and summary graph in (C) show cytoplasmic MPO staining in WT and *Il23a*^-/-^ bone marrow neutrophils and in a CD45^+^CD11b^-^ negative control population (ctrl). Bars are the mean + SD of n = 3. (D) WT and *Il23a*^-/-^ bone marrow neutrophils were co-incubated with *C*. *albicans* hyphae at a 20:1 ratio. The percentage of *C*. *albicans* killing was assessed using WST-1 reagent. Bars are the mean with SD of each group with n  =  4. (E—F) WT and *Il23a*^*-/-*^ mice were infected intravenously with 2x10^5^ CFU *C*. *albicans* and neutrophil morphology in the kidney was quantified by flow cytometry 24h post infection. Representative FACS plots in (E) and summary graphs in (F) show the side scatter (SSC), which gives an indication of the cell's granularity, and the forward scatter (FSC), which correlates with the size of the cell. Bars are the mean + SD of each group with n = 3. Data A and D—F are representative of two independent experiments. Statistics were calculated using unpaired Student’s t-Test. ****p<0.0001.
(TIF)

S5 Fig Infection with a yeast-locked *C*.*albicans* mutant or *in vitro* co-culture with *C*. *albicans* or fungal PAMPs does not impair viability of neutrophils from IL-23 pathway-deficient mice. (A, B) WT and *Il23r*^gfp/gfp^ mice were infected intravenously with 3x10^5^ CFU yeast-locked mutant *C*. *albicans* strain *hgc1*Δ/Δ. (A) Myeloid cell populations in the kidney were quantified by flow cytometry at 48h post infection. Neutrophils, Ly6C^hi^ monocytes and Ly6C^lo^ myeloid cells were defined as shown in Fig 2C. Summary graphs show the absolute numbers of each cell population per kidney. Each dot represents one animal and the mean of each group is indicated. (B) Viability of neutrophils was assessed by flow cytometry at 48h post infection using 7-AAD and Annexin V reagents as described in Fig 4A. Summary graphs show the percentage of 7-AAD^-^Annexin V^-^, 7-AAD^-^Annexin V^+^ and 7-AAD^+^Annexin V^+^ populations among total neutrophils. Bars are the mean + SD of each group with n = 4. (C) Neutrophils purified from the bone marrow of naïve WT and *Il23r*^gfp/gfp^ mice were left unstimulated (ctrl) or were co-cultured with curdlan, heat-killed *C*. *albicans* hyphae (h.k. *C*. *albicans*), viable *C*. *albicans* hyphae (*C*. *albicans*). Viability of neutrophils was assessed by flow cytometry using 7-AAD and Annexin V reagents. Summary graphs show the percentage of 7-AAD^-^Annexin V^-^, 7-AAD^-^Annexin V^+^ and 7-AAD^+^Annexin V^+^ populations among total neutrophils that were pre-gated as shown in Fig 2C without prior exclusion of dead cells. Bars are the mean + SD of each group with n = 3. Statistics were calculated using unpaired Student’s t-Test. **p<0.01.
(TIF)

S6 Fig Epicutaneous *M*. *pachydermatis* infection does not impair myeloid cell viability in *Il23r*^gfp/gfp^ mice. **WT and *Il23r***^**gfp/gfp**^ **mice were infected with 2 OD**_**600**_ **of *M*. *pachydermatis* after mild tape stripping of the dorsal ear skin.** (A) Viability of neutrophils was assessed by flow cytometry at 48h post infection using 7-AAD and Annexin V reagents as described in Fig 4A. Summary graphs show the percentage of 7-AAD^-^Annexin V^-^, 7-AAD^-^Annexin V^+^ and 7-AAD^+^Annexin V^+^ populations among total neutrophils. Bars are the mean + SD of each group with n = 4. (B) Myeloid cell populations in the ear were quantified by flow cytometry at 48h post infection. Neutrophils, Ly6C^hi^ monocytes and Ly6C^lo^ myeloid cells were defined as shown in Fig 2C. Summary graphs show the absolute numbers of each cell population per ear. Each dot represents one animal and the mean of each group is indicated. Statistics were calculated using unpaired Student’s t-Test. **p<0.01.
(TIF)

S7 Fig Viability of kidney neutrophils after enrichment by density gradient centrifugation from WT and *Il23a*^*-/-*^ mice at 24h post infection is comparable and apoptosis is the prevalent form of cell death in neutrophils and Ly6C^hi^ monocytes from the kidney of *Il23a*^*-/-*^ mice. WT and *Il23a*^*-/-*^ mice were infected intravenously with 2x10^5^ CFU *C*. *albicans*. Kidney myeloid cells (A, C) and BM neutrophils (B) were isolated by density gradient centrifugation at 24h post infection. (A) Neutrophil viability was assessed by flow cytometry using 7-AAD and Annexin V reagents directly after density gradient centrifugation. Representative FACS plots of neutrophils that were pre-gated as shown in Fig 2C without prior exclusion of dead cells. (B) The release of extracellular DNA from isolated BM neutrophils was detected by Sytox green after stimulation for 2.5 h with preformed *C*. *albicans* hyphae. The increase in fluorescence intensity from stimulated relative to unstimulated neutrophils is shown. Bars are the mean + SD of each group with n = 4. (C) Kidney myeloid cells were cultured with Q-VD-OPh or DMSO as a control in supplemented RPMI 1640 medium for 18h. The cell viability was then assessed as described in (A). Summary graphs show the percentage of 7-AAD^-^Annexin V^-^ and 7-AAD^+^Annexin V^+^ populations among the total population of the respective myeloid subset. Bars are the mean + SD of each group with n = 4. Data are representative of two independent experiments. Statistics were calculated using unpaired Student’s t-Test. *p<0.05, **p<0.01, ***p<0.001, ****p<0.0001.
(TIF)

S8 Fig IL-23 promotes the viability of myeloid cells independently of lymphoid cells, IL-17 and GM-CSF. (A—D) WT, *Il17ra*^*-/-*^, *Csf2*^*-/-*^ and *Rag2*^*-/-*^*Il2rg*^*-/-*^ mice were infected intravenously with 2x10^5^ CFU *C*. *albicans*. (A, C, D) Viability of kidney neutrophils was assessed by flow cytometry at 48h post infection using (A, D) 7-AAD and Annexin V reagents as described in Fig 4A or (C) LIVE/DEAD Fixable Near-IR Dead Cell Stain Kit. Summary graphs show the percentage of 7-AAD^-^Annexin V^-^ and 7-AAD^+^Annexin V^+^ populations among total neutrophils, Ly6C^hi^ monocytes and Ly6C^lo^ myeloid cells. Neutrophils, Ly6C^hi^ monocytes and Ly6C^lo^ myeloid cells were defined as shown in Fig 2C. Bars are the mean + SD of each group with n = 3 (except for the WT group in D for which n = 2). Data are representative of two independent experiments. (B) Ly6C^lo^ myeloid cells in the kidney of WT and *Il17ra*^*-/-*^ mice were quantified by flow cytometry at 48h post infection. Each dot represents one animal and the mean of each group is indicated. Statistics were calculated using unpaired Student’s t-Test. *p<0.05, **p<0.01.
(TIF)

S9 Fig IL-23 promotes the viability of myeloid cells in a non-cell intrinsic manner. (A—C) WT (CD45.1)/*Il23r*^gfp/gfp^ (CD45.2) mixed BM chimeras mice were infected intravenously with 3x10^5^ CFU *C*. *albicans*. (A) Representative FACS plots showing the percentage of the WT (CD45.1^+^) and *Il23r*^gfp/gfp^ (CD45.2^+^) neutrophils among total blood neutrophils in naïve mice (left) and among total kidney neutrophils at 48h post infection (right). Neutrophils were pre-gated as shown in Fig 2C. (B) Summary graphs showing the WT:*Il23r*^gfp/gfp^ ratio of myeloid cells in the blood of naïve chimeras (left) and in the kidney of infected chimeras at 48h post infection (right). Neutrophils, Ly6C^hi^ monocytes and Ly6C^lo^ myeloid cells were defined as shown in Fig 2C. Each dot represents one animal and the mean of each group is indicated. Data are representative of two independent experiments. (C) Viability of neutrophils, Ly6C^hi^ monocytes and Ly6C^lo^ myeloid cells in the WT (CD45.1^+^) and *Il23r*^gfp/gfp^ (CD45.2^+^) compartment was assessed by flow cytometry at 48h post infection using 7-AAD and Annexin V reagents as described in Fig 4A. Summary graphs show the percentage of 7-AAD^-^Annexin V^-^ and 7-AAD^+^Annexin V^+^ populations among total cells. Bars are the mean + SD of each group with n = 5. Data are representative of two independent experiments. Statistics were calculated using unpaired Student’s t-Test. *p<0.05.
(TIF)

S10 Fig Neutrophils and Ly6C^hi^ monocytes are the primary source of IL-23 during systemic candidiasis. Refined analysis of the data shown in Fig 5A from naïve and *C*. *albicans*-infected WT mice. Representative FACS plots show *Il23a* mRNA expression in neutrophils, Ly6C^hi^ monocytes and Ly6C^lo^ myeloid cells. Neutrophils, Ly6C^hi^ monocytes and Ly6C^lo^ myeloid cells were defined as shown in Fig 2C. A *C*. *albicans*-infected sample, in which the *Il23r* gene-specific target probe set was omitted, served as a control (ctrl).
(TIF)

S1 Table Chemicals, reagents, antibodies, mouse strains, fungal and bacterial strains, instruments and software used in the study. (DOCX)
