# Supplementary figures and images for: IL-23 supports host defense against systemic Candida albicans infection by ensuring myeloid cell survival
Source: PLoS Pathog. 2019 Dec 30;15(12):e1008115. doi: 10.1371/journal.ppat.1008115 (PMC6957211; doi:10.1371/journal.ppat.1008115)

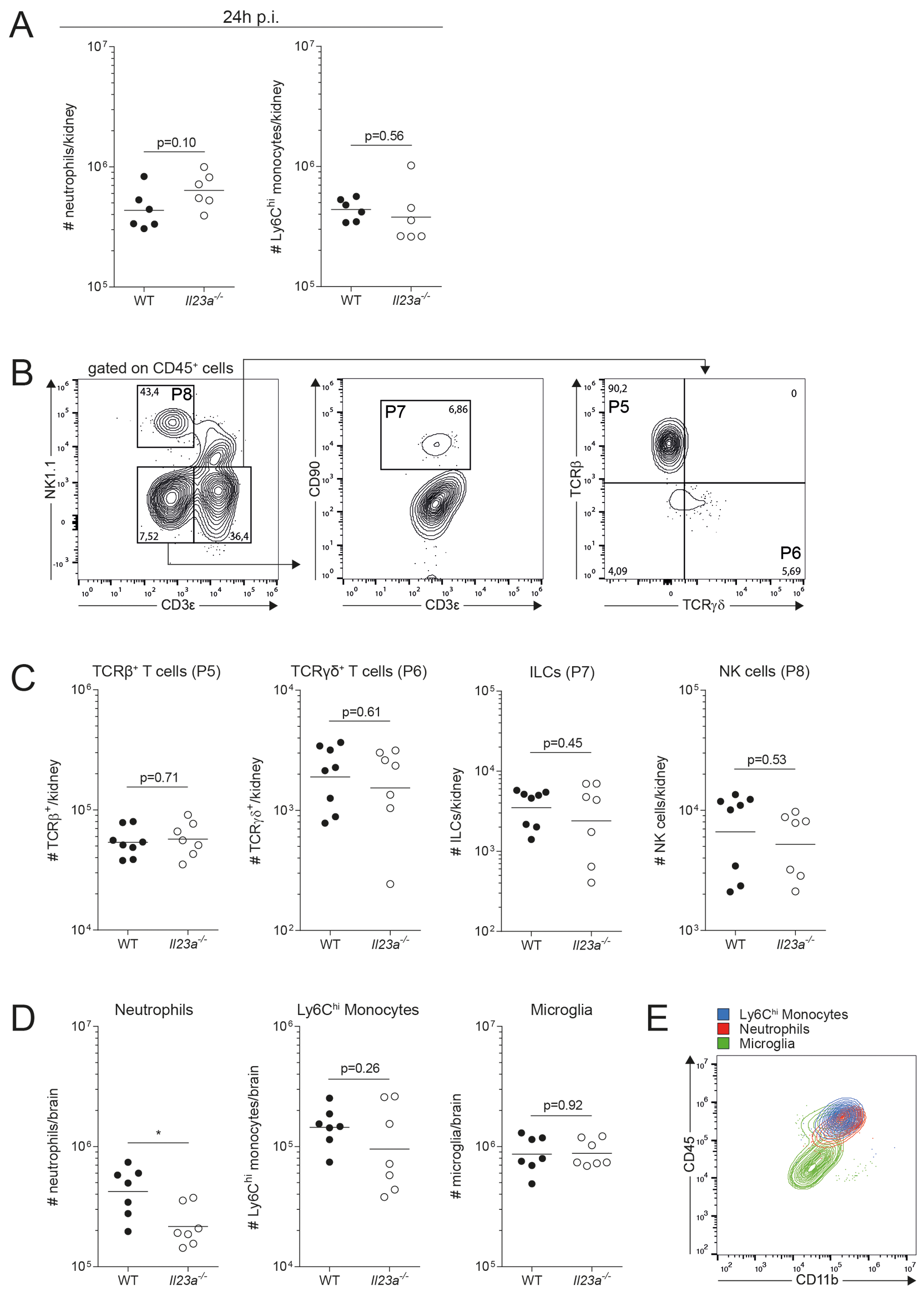

Supplement: S1 Fig — WT and Il23a-/- mice were infected intravenously with 2x10^5^ CFU C. albicans. (A) Neutrophils and Ly6Chi monocytes were quantified by flow cytometry at 24h post infection. (B-C) Lymphoid cell populations in the kidney were quantified by flow cytometry 48h post infection. Representative FACS plots in (B) show the gating strategy for kidney lymphoid cells. TCRβ+ T cells (P5), TCRγδ+ T cells (P6), ILCs (P7) and NK cells (P8) were defined as indicated. Summary graphs in (C) show the absolute numbers of each cell population per kidney. (D) Myeloid cell populations in the brain were quantified by flow cytometry at 48h post infection. Neutrophils and Ly6Chi monocytes were defined as shown in Fig 2C. Ly6Clo myeloid cells in the brain correspond by the large majority to microglia, which are defined by their low expression of CD45. (E) Representative FACS plot showing CD11b and CD45 expression in neutrophils, Ly6Chi monocytes and microglia, gated as described in (D). In A, C and D, each dot represents one animal and the mean of each group is indicated. Data are pooled from two independent experiments. Statistics were calculated using unpaired Student’s t-Test. *p<0.05. (TIF) [file ppat.1008115.s001.tif]

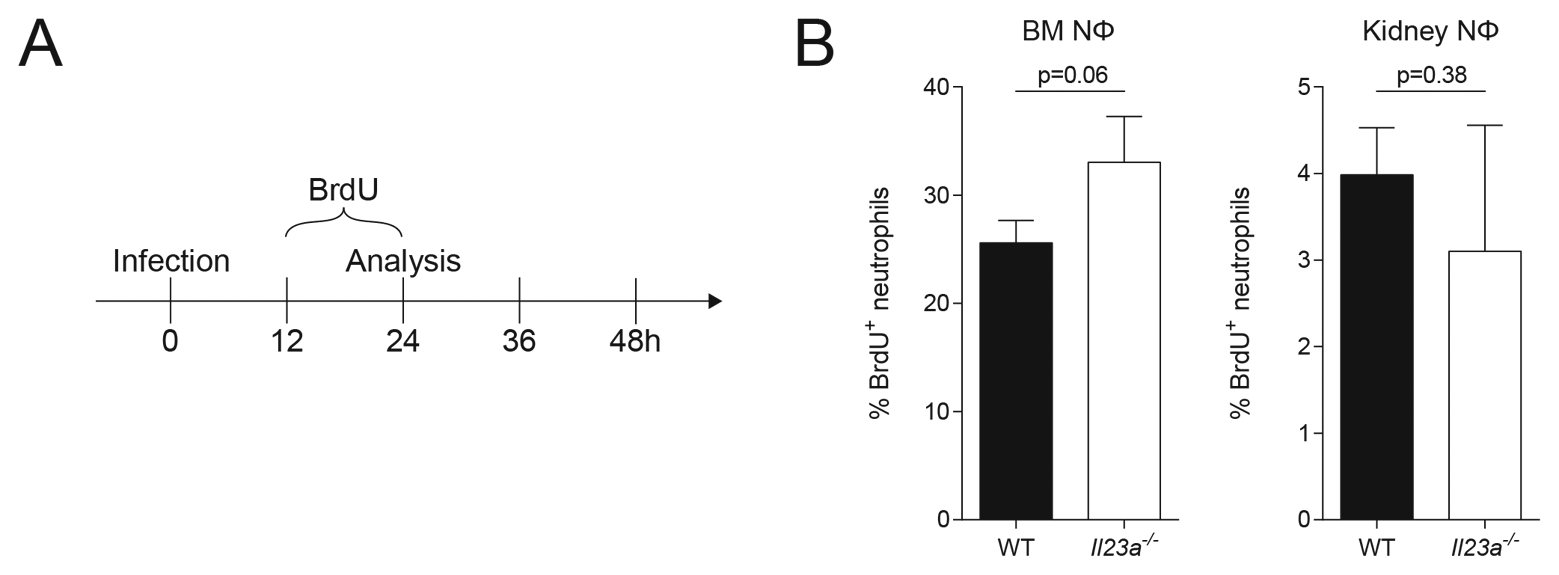

Supplement: S2 Fig — WT and Il23a-/- mice were infected intravenously with 2x10^5^ CFU C. albicans. Mice were treated with BrdU starting from 12h post infection and neutrophil proliferation was assessed 24h post infection. (A) Schematic representation of experimental design. (B) Summary graphs show percentage of BrdU+ cells within the total population of BM neutrophils (left) or kidney neutrophils (right). Neutrophils were defined as shown in Fig 2C. Bars are the mean + SD of each group with n = 3. Statistics were calculated using unpaired Student’s t-Test. (TIF) [file ppat.1008115.s002.tif]

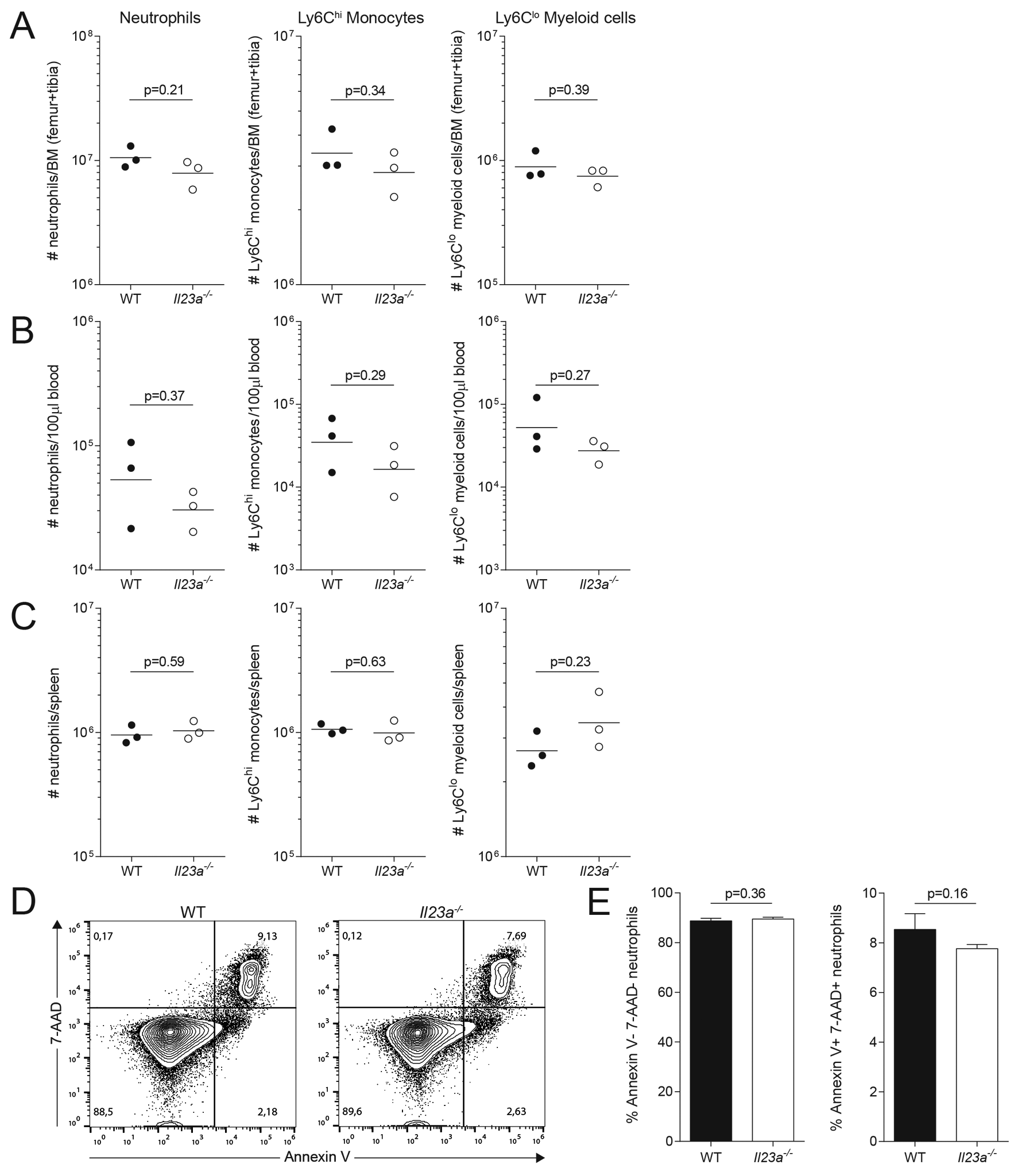

Supplement: S3 Fig — (A-C) Myeloid cell populations in (A) bone marrow, (B) blood and (C) spleen of naive WT and Il23a-/- mice were quantified by flow cytometry. Neutrophils, Ly6Chi monocytes and Ly6Clo myeloid cells were defined as shown in Fig 2C. Summary graphs show the absolute numbers of each cell population per kidney. Each dot represents one animal and the mean of each group is indicated. (D, E) Neutrophils were purified from the bone marrow of naïve WT and Il23a-/- mice and cultured in supplemented RPMI 1640 medium for 18h. Viability of neutrophils was assessed by flow cytometry using 7-AAD and Annexin V reagents. (D) Representative FACS plots of neutrophils that were pre-gated on neutrophils as shown in Fig 2C without prior exclusion of dead cells. (E) Summary graphs show the percentage of 7-AAD-Annexin V- and 7-AAD+Annexin V+ populations among total neutrophils. Bars are the mean + SD of each group with n = 3. Statistics were calculated using unpaired Student’s t-Test. (TIF) [file ppat.1008115.s003.tif]

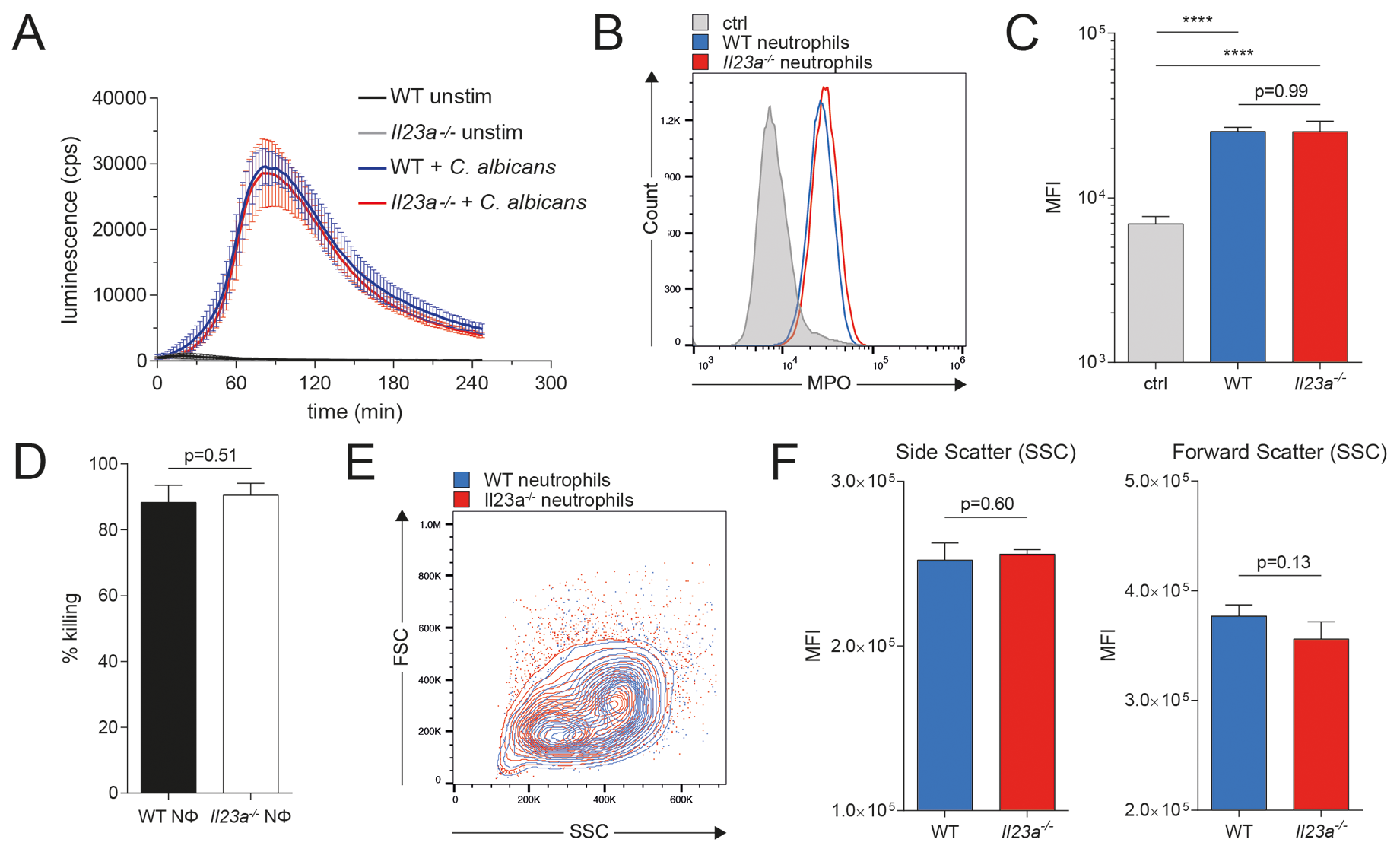

Supplement: S4 Fig — (A) Reactive oxygen species (ROS) production by WT and Il23a-/- bone marrow neutrophils in response to C. albicans yeast was detected by chemiluminescence using luminol reagent. Curves are the mean + SD of each group with n = 3. (B—C) Representative histogram in (B) and summary graph in (C) show cytoplasmic MPO staining in WT and Il23a-/- bone marrow neutrophils and in a CD45+CD11b- negative control population (ctrl). Bars are the mean + SD of n = 3. (D) WT and Il23a-/- bone marrow neutrophils were co-incubated with C. albicans hyphae at a 20:1 ratio. The percentage of C. albicans killing was assessed using WST-1 reagent. Bars are the mean with SD of each group with n = 4. (E—F) WT and Il23a-/- mice were infected intravenously with 2x10^5^ CFU C. albicans and neutrophil morphology in the kidney was quantified by flow cytometry 24h post infection. Representative FACS plots in (E) and summary graphs in (F) show the side scatter (SSC), which gives an indication of the cell's granularity, and the forward scatter (FSC), which correlates with the size of the cell. Bars are the mean + SD of each group with n = 3. Data A and D—F are representative of two independent experiments. Statistics were calculated using unpaired Student’s t-Test. ****p<0.0001. (TIF) [file ppat.1008115.s004.tif]

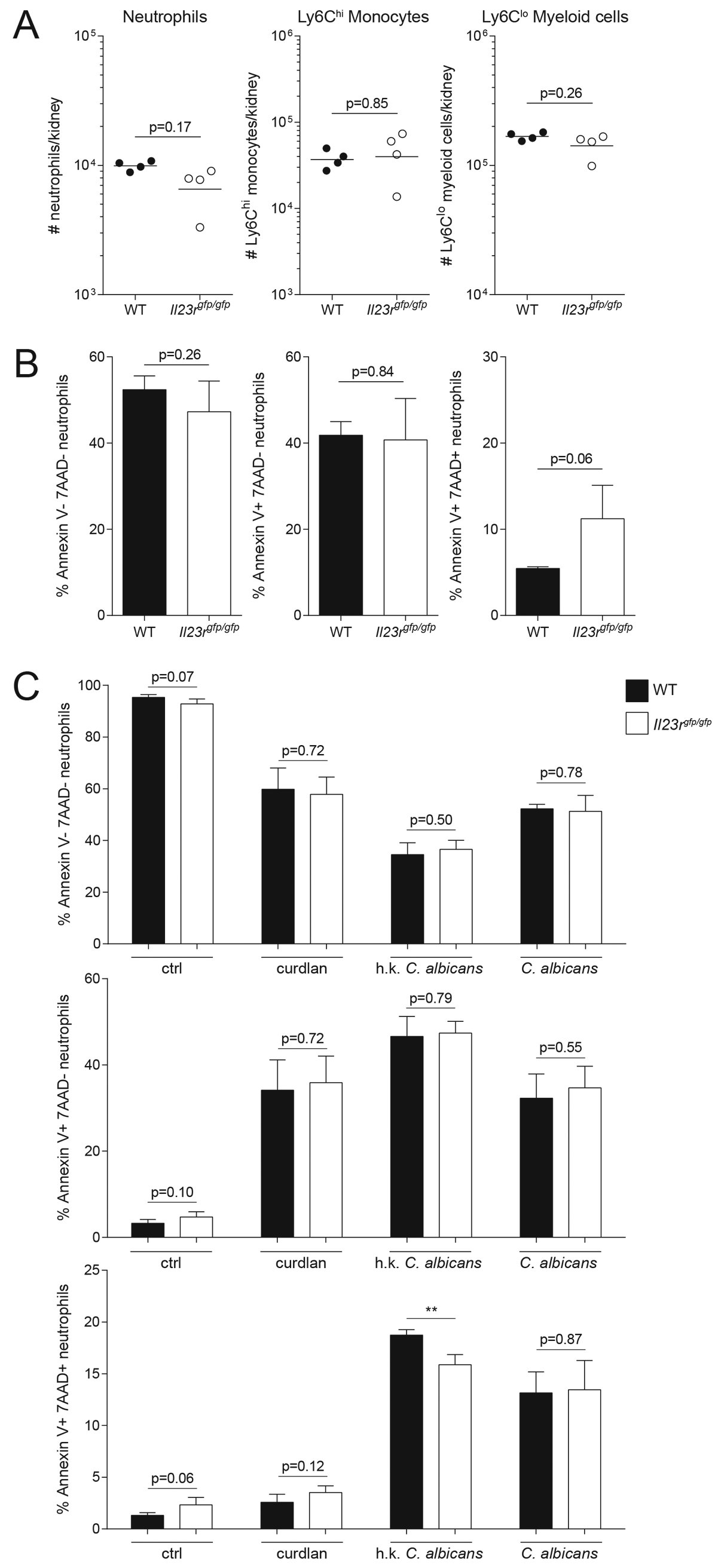

Supplement: S5 Fig — (A, B) WT and Il23rgfp/gfp mice were infected intravenously with 3x10^5^ CFU yeast-locked mutant C. albicans strain hgc1Δ/Δ. (A) Myeloid cell populations in the kidney were quantified by flow cytometry at 48h post infection. Neutrophils, Ly6Chi monocytes and Ly6Clo myeloid cells were defined as shown in Fig 2C. Summary graphs show the absolute numbers of each cell population per kidney. Each dot represents one animal and the mean of each group is indicated. (B) Viability of neutrophils was assessed by flow cytometry at 48h post infection using 7-AAD and Annexin V reagents as described in Fig 4A. Summary graphs show the percentage of 7-AAD-Annexin V-, 7-AAD-Annexin V+ and 7-AAD+Annexin V+ populations among total neutrophils. Bars are the mean + SD of each group with n = 4. (C) Neutrophils purified from the bone marrow of naïve WT and Il23rgfp/gfp mice were left unstimulated (ctrl) or were co-cultured with curdlan, heat-killed C. albicans hyphae (h.k. C. albicans), viable C. albicans hyphae (C. albicans). Viability of neutrophils was assessed by flow cytometry using 7-AAD and Annexin V reagents. Summary graphs show the percentage of 7-AAD-Annexin V-, 7-AAD-Annexin V+ and 7-AAD+Annexin V+ populations among total neutrophils that were pre-gated as shown in Fig 2C without prior exclusion of dead cells. Bars are the mean + SD of each group with n = 3. Statistics were calculated using unpaired Student’s t-Test. **p<0.01. (TIF) [file ppat.1008115.s005.tif]

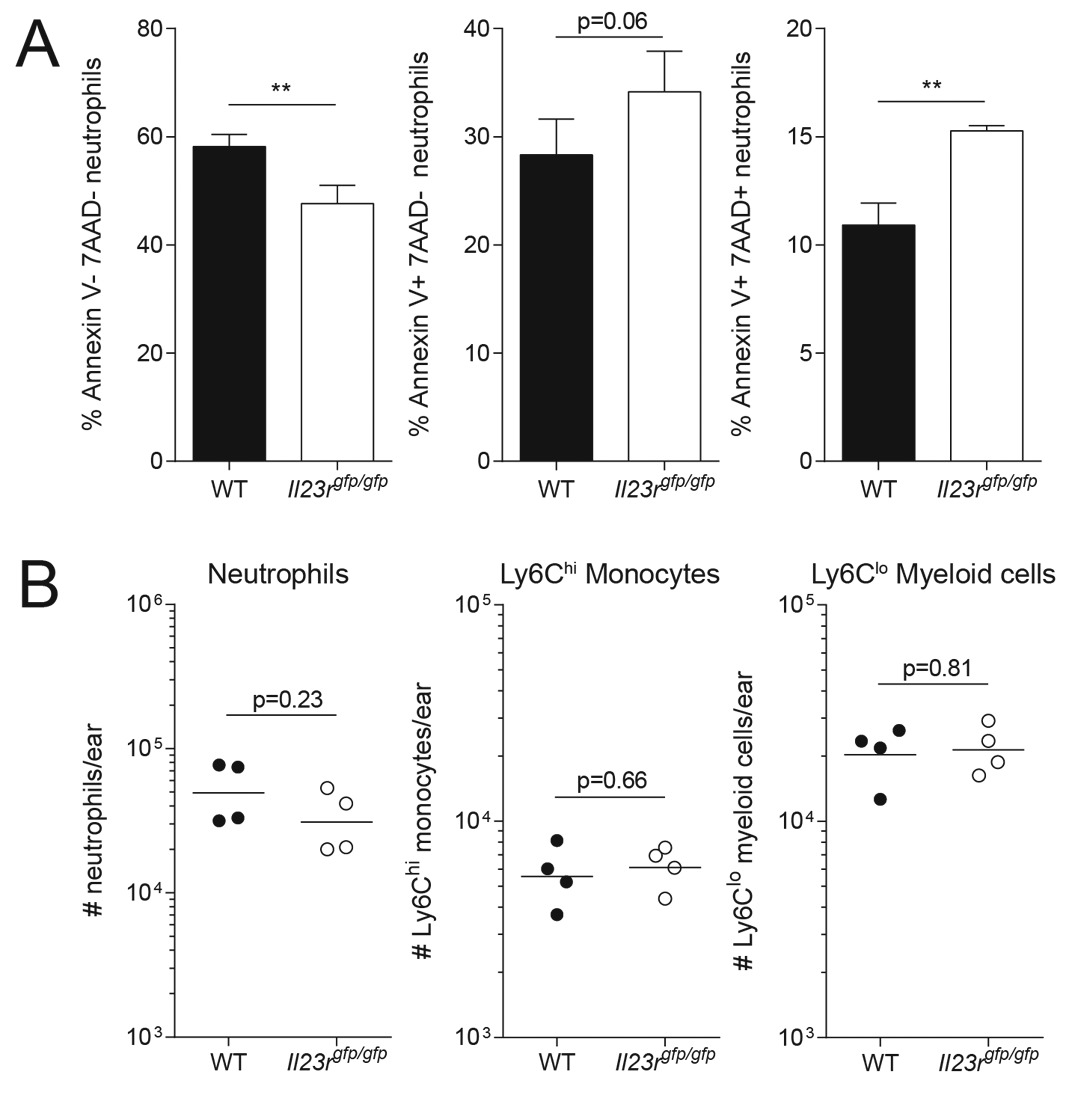

Supplement: S6 Fig — WT and Il23rgfp/gfp mice were infected with 2 OD600 of M. pachydermatis after mild tape stripping of the dorsal ear skin. (A) Viability of neutrophils was assessed by flow cytometry at 48h post infection using 7-AAD and Annexin V reagents as described in Fig 4A. Summary graphs show the percentage of 7-AAD-Annexin V-, 7-AAD-Annexin V+ and 7-AAD+Annexin V+ populations among total neutrophils. Bars are the mean + SD of each group with n = 4. (B) Myeloid cell populations in the ear were quantified by flow cytometry at 48h post infection. Neutrophils, Ly6Chi monocytes and Ly6Clo myeloid cells were defined as shown in Fig 2C. Summary graphs show the absolute numbers of each cell population per ear. Each dot represents one animal and the mean of each group is indicated. Statistics were calculated using unpaired Student’s t-Test. **p<0.01. (TIF) [file ppat.1008115.s006.tif]

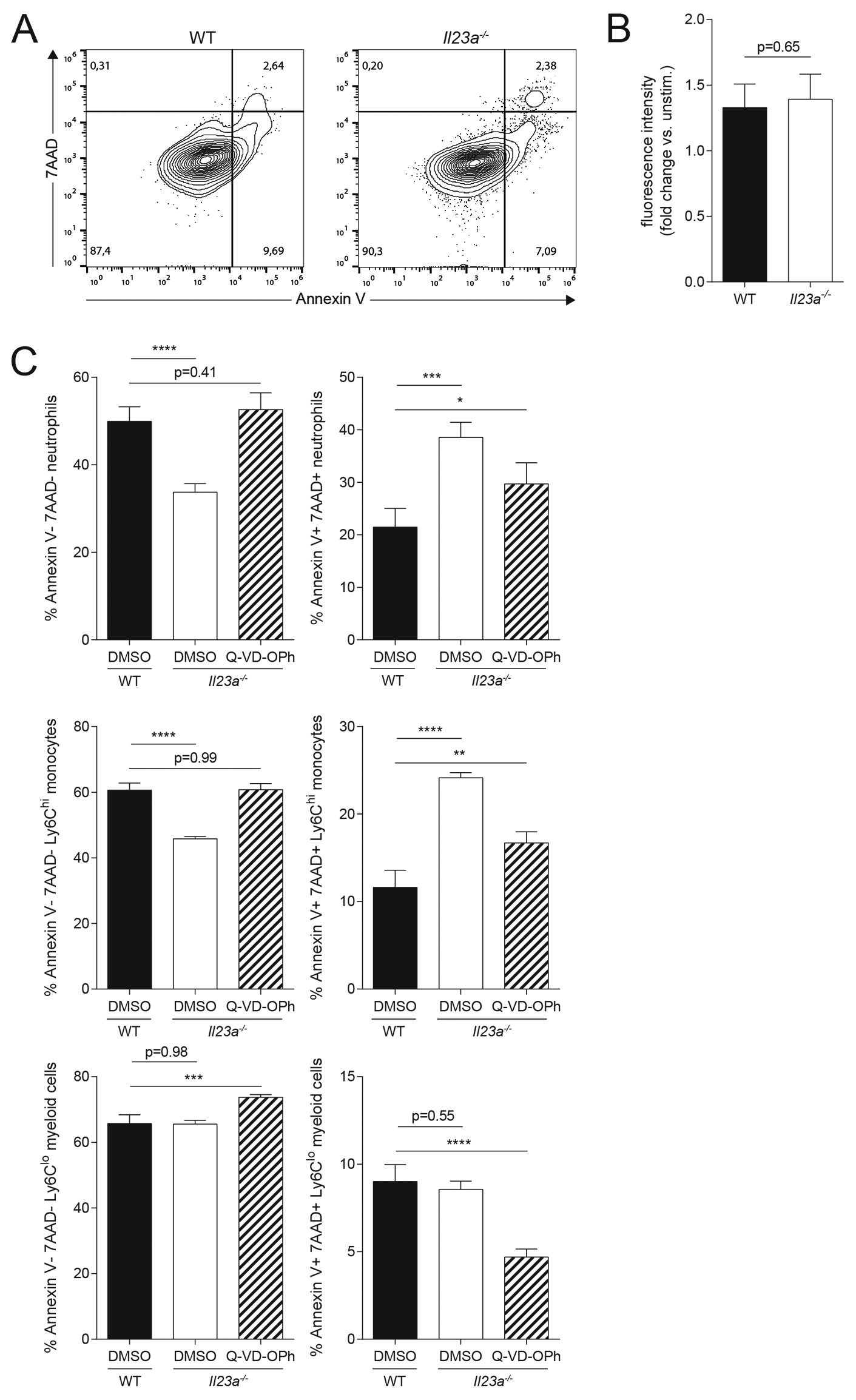

Supplement: S7 Fig — WT and Il23a-/- mice were infected intravenously with 2x10^5^ CFU C. albicans. Kidney myeloid cells (A, C) and BM neutrophils (B) were isolated by density gradient centrifugation at 24h post infection. (A) Neutrophil viability was assessed by flow cytometry using 7-AAD and Annexin V reagents directly after density gradient centrifugation. Representative FACS plots of neutrophils that were pre-gated as shown in Fig 2C without prior exclusion of dead cells. (B) The release of extracellular DNA from isolated BM neutrophils was detected by Sytox green after stimulation for 2.5 h with preformed C. albicans hyphae. The increase in fluorescence intensity from stimulated relative to unstimulated neutrophils is shown. Bars are the mean + SD of each group with n = 4. (C) Kidney myeloid cells were cultured with Q-VD-OPh or DMSO as a control in supplemented RPMI 1640 medium for 18h. The cell viability was then assessed as described in (A). Summary graphs show the percentage of 7-AAD-Annexin V- and 7-AAD+Annexin V+ populations among the total population of the respective myeloid subset. Bars are the mean + SD of each group with n = 4. Data are representative of two independent experiments. Statistics were calculated using unpaired Student’s t-Test. *p<0.05, **p<0.01, ***p<0.001, ****p<0.0001. (TIF) [file ppat.1008115.s007.tif]

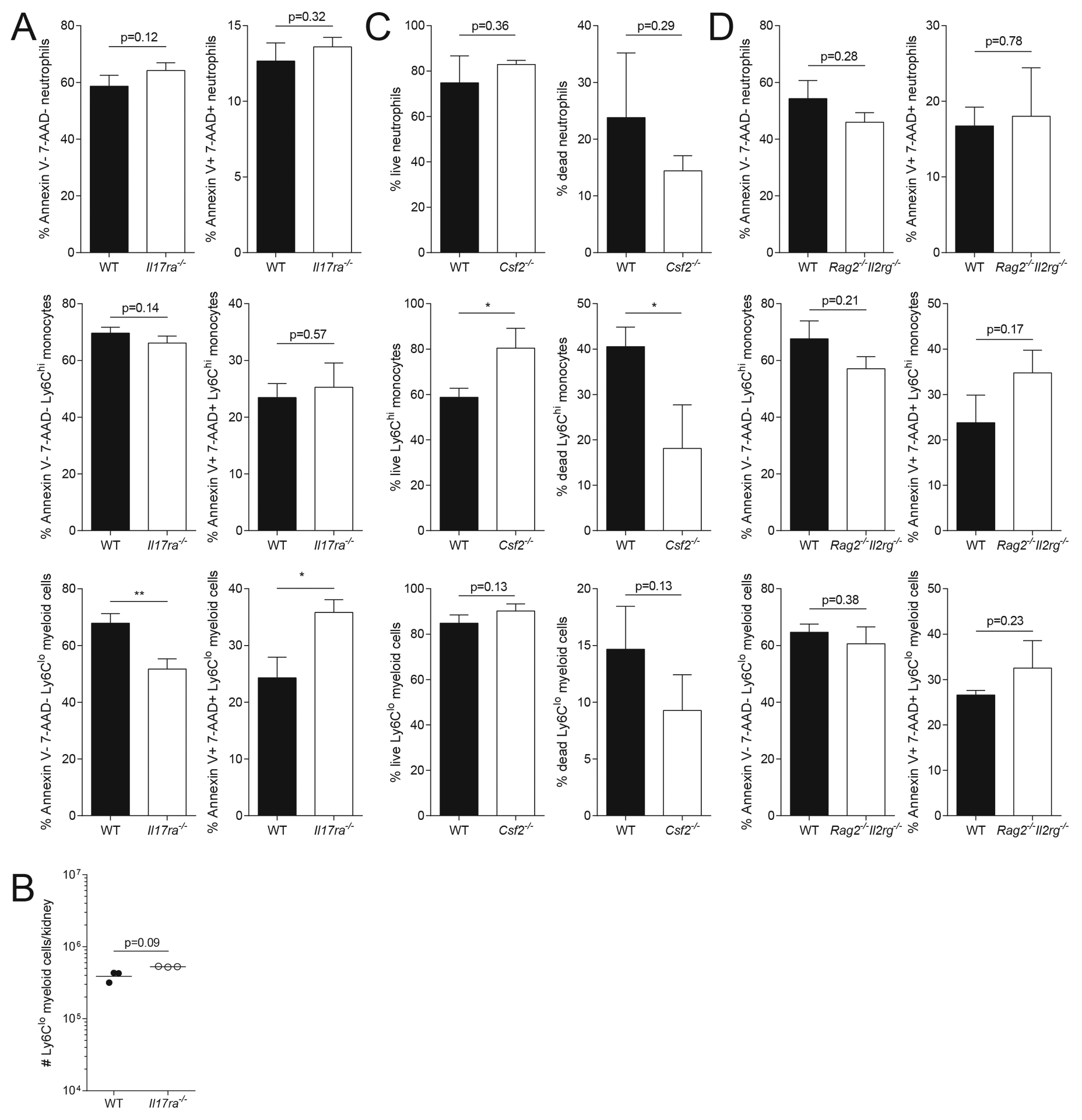

Supplement: S8 Fig — (A—D) WT, Il17ra-/-, Csf2-/- and Rag2-/-Il2rg-/- mice were infected intravenously with 2x10^5^ CFU C. albicans. (A, C, D) Viability of kidney neutrophils was assessed by flow cytometry at 48h post infection using (A, D) 7-AAD and Annexin V reagents as described in Fig 4A or (C) LIVE/DEAD Fixable Near-IR Dead Cell Stain Kit. Summary graphs show the percentage of 7-AAD-Annexin V- and 7-AAD+Annexin V+ populations among total neutrophils, Ly6Chi monocytes and Ly6Clo myeloid cells. Neutrophils, Ly6Chi monocytes and Ly6Clo myeloid cells were defined as shown in Fig 2C. Bars are the mean + SD of each group with n = 3 (except for the WT group in D for which n = 2). Data are representative of two independent experiments. (B) Ly6Clo myeloid cells in the kidney of WT and Il17ra-/- mice were quantified by flow cytometry at 48h post infection. Each dot represents one animal and the mean of each group is indicated. Statistics were calculated using unpaired Student’s t-Test. *p<0.05, **p<0.01. (TIF) [file ppat.1008115.s008.tif]

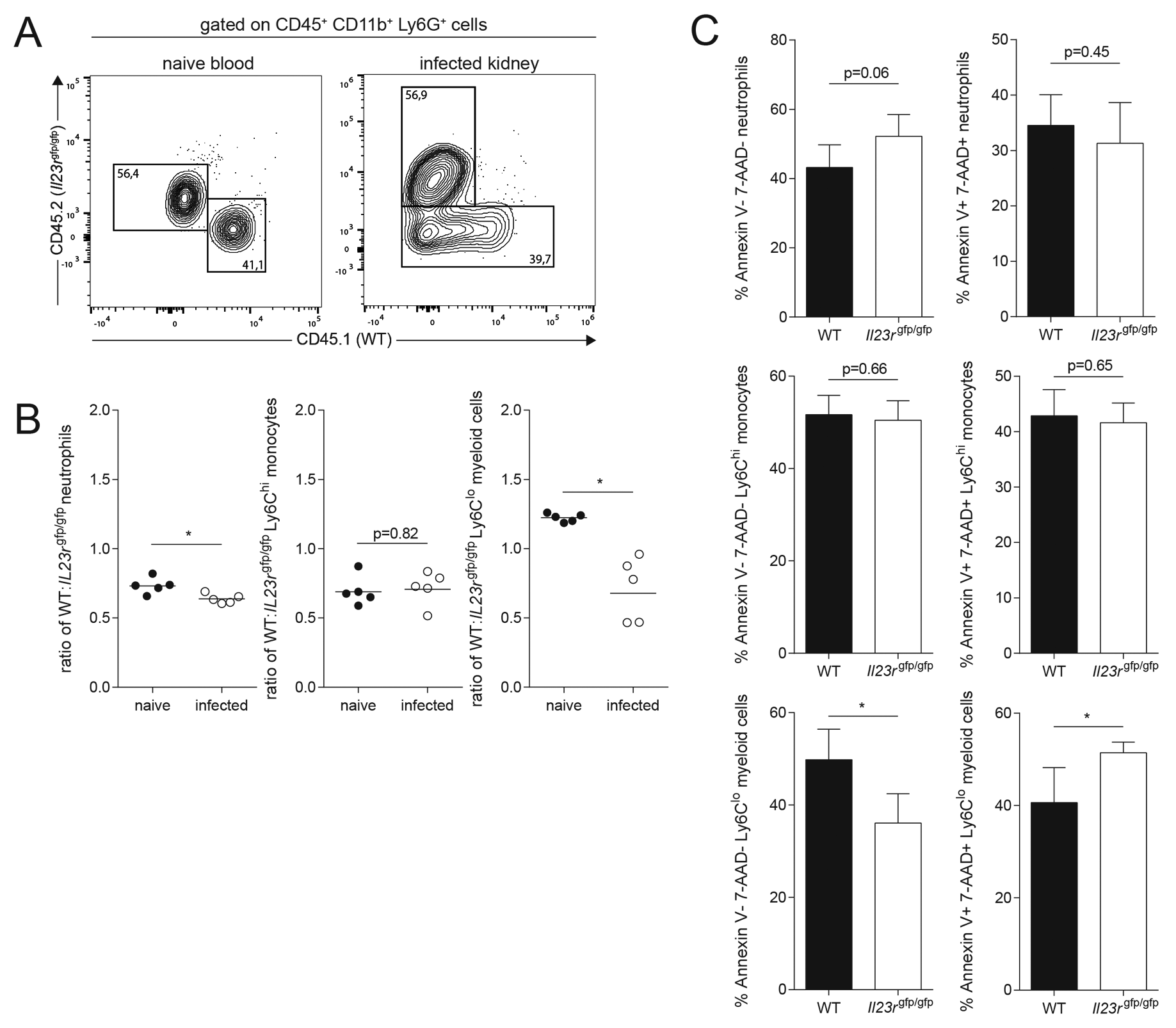

Supplement: S9 Fig — (A—C) WT (CD45.1)/Il23rgfp/gfp (CD45.2) mixed BM chimeras mice were infected intravenously with 3x10^5^ CFU C. albicans. (A) Representative FACS plots showing the percentage of the WT (CD45.1+) and Il23rgfp/gfp (CD45.2+) neutrophils among total blood neutrophils in naïve mice (left) and among total kidney neutrophils at 48h post infection (right). Neutrophils were pre-gated as shown in Fig 2C. (B) Summary graphs showing the WT:Il23rgfp/gfp ratio of myeloid cells in the blood of naïve chimeras (left) and in the kidney of infected chimeras at 48h post infection (right). Neutrophils, Ly6Chi monocytes and Ly6Clo myeloid cells were defined as shown in Fig 2C. Each dot represents one animal and the mean of each group is indicated. Data are representative of two independent experiments. (C) Viability of neutrophils, Ly6Chi monocytes and Ly6Clo myeloid cells in the WT (CD45.1+) and Il23rgfp/gfp (CD45.2+) compartment was assessed by flow cytometry at 48h post infection using 7-AAD and Annexin V reagents as described in Fig 4A. Summary graphs show the percentage of 7-AAD-Annexin V- and 7-AAD+Annexin V+ populations among total cells. Bars are the mean + SD of each group with n = 5. Data are representative of two independent experiments. Statistics were calculated using unpaired Student’s t-Test. *p<0.05. (TIF) [file ppat.1008115.s009.tif]

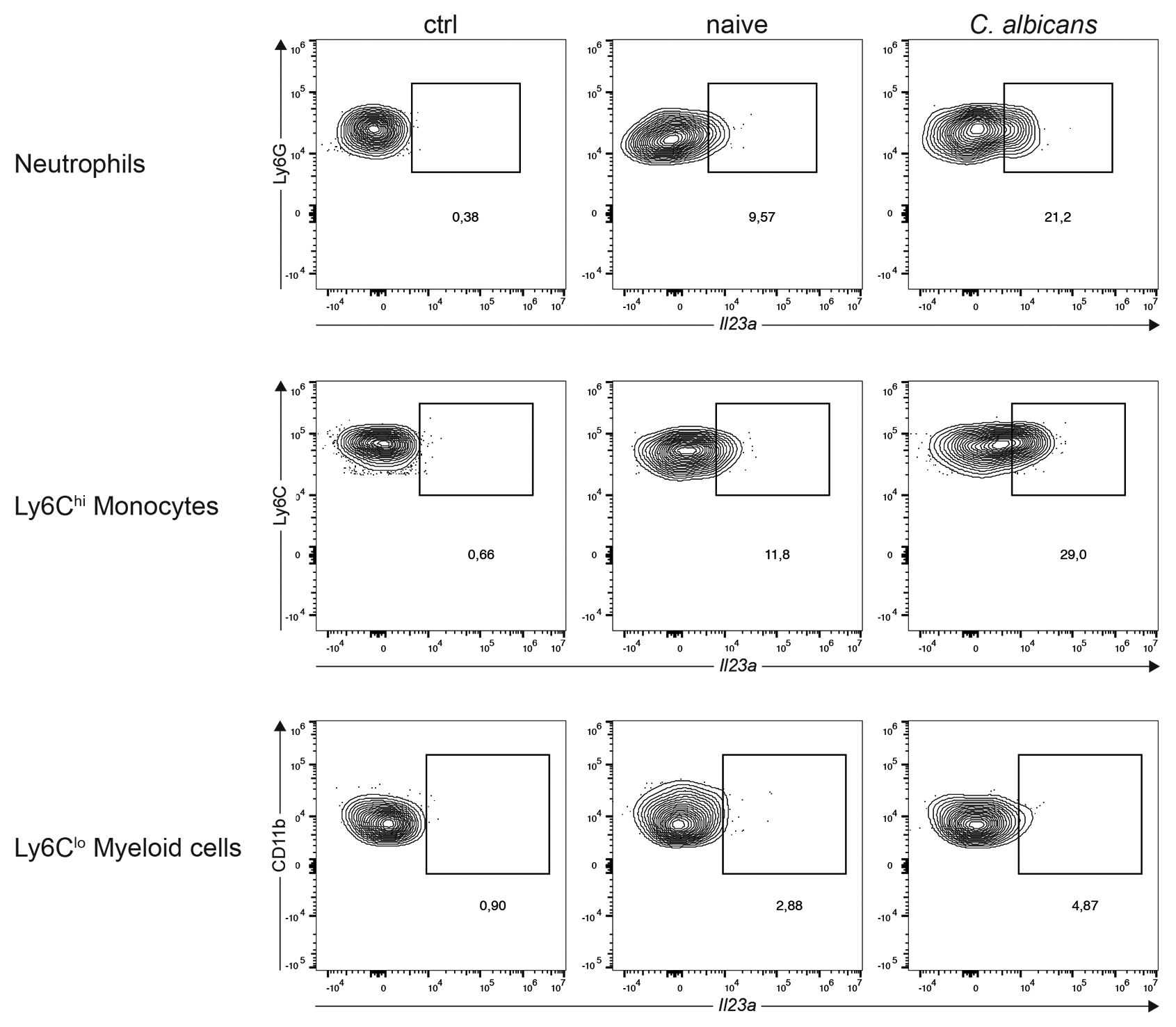

Supplement: S10 Fig — Refined analysis of the data shown in Fig 5A from naïve and C. albicans-infected WT mice. Representative FACS plots show Il23a mRNA expression in neutrophils, Ly6Chi monocytes and Ly6Clo myeloid cells. Neutrophils, Ly6Chi monocytes and Ly6Clo myeloid cells were defined as shown in Fig 2C. A C. albicans-infected sample, in which the Il23r gene-specific target probe set was omitted, served as a control (ctrl). (TIF) [file ppat.1008115.s010.tif]
