# Supplementary material for: IL-23 supports host defense against systemic Candida albicans infection by ensuring myeloid cell survival
Source: PLoS Pathog. 2019 Dec 30;15(12):e1008115. doi: 10.1371/journal.ppat.1008115 (PMC6957211; doi:10.1371/journal.ppat.1008115)
Supplement: S1 Table — (DOCX) [file ppat.1008115.s011.docx]

**Supplementary Table S1:**

| **REAGENT or RESOURCE** | **SOURCE** | **IDENTIFIER** |
| --- | --- | --- |
| **Antibodies (clones)** | | |
| PE/Cy5 anti-CD45 (30-F11) | Biolegend | Cat# 103109 |
| APC anti-CD45.2 (104) | Biolegend | Cat# 109814 |
| Pacific Blue anti-CD45.2 (104) | Biolegend | Cat# 109820 |
| Pacific Blue anti-CD45.1 (A20) | Biolegend | Cat# 110722 |
| FITC anti-CD11b (M1/70) | eBioscience | Cat# 11-0112 |
| PE/Cy7 anti-CD11b (M1/70) | Biolegend | Cat# 101216 |
| BV711 anti-CD11b (M1/70) | Biolegend | Cat# 101241 |
| APC anti-CD11c (N418) | Biolegend | Cat# 117310 |
| PE/Cy7 anti-CD11c (N418) | Biolegend | Cat# 117318 |
| PerCP anti-MHCII (M5/114.15.2) | Biolegend | Cat# 107624 |
| Pacific Blue anti-Ly6G (1A8) | Biolegend | Cat# 127612 |
| FITC anti-Ly6G (1A8) | Biolegend | Cat# 127605 |
| BV570 anti-Ly6C (HK1.4) | Biolegend | Cat# 128029 |
| APC anti-F4/80 (BM8) | Biolegend | Cat# 123116 |
| PE anti-F4/80 (BM8) | Biolegend | Cat# 123109 |
| FITC anti-CD3e (145-2C11) | Biolegend | Cat# 100306 |
| PE/Cy5 anti-CD3e (145-2C11) | Biolegend | Cat# 100310 |
| PE anti-NK1.1 (PK136) | Biolegend | Cat# 108708 |
| BV570 anti-CD90.2 (30-H12) | Biolegend | Cat# 105329 |
| APC anti-TCRβ (H57-597) | Biolegend | Cat# 109212 |
| PerCP/Cy5.5 anti-TCRγδ (GL3) | Biolegend | Cat# 118118 |
| FITC anti-MPO (8F4) | HycultBiotech | Cat# HM1051F |
| anti-G-CSF (67604) | R&D Systems | Cat# RB01 |
| anti-G-CSF (polyclonal) | Peprotech | Cat# 250-05 |
| anti-Cleaved Caspase-3 (Asp175) | Cell Signaling Technology | Cat# 9664T |
| anti-CD11b (M1/70) | Biolegend | Cat# 101201 |
| Anti-Rat IgG (H+L) (polyclonal) | Jackson ImmunoResearch | Cat# 112-165-003 |
| Anti-Rabbit IgG (H+L) (polyclonal) | Jackson ImmunoResearch | Cat# 111-605-144 |
| **Fungal and bacterial strains** | | |
| *C. albicans* | [62] | Strain SC5314 |
| *C. albicans* | [31] | Strain *hgc1*Δ/Δ |
| *S. aureus* | Annelies Zinkgernagel, University of Zurich [63] | Strain Newman, ATCC 25904 |
| *M. pachydermatis* | [64] | Strain ATCC 14522 |
| **Biological samples** | | |
| Fetal Calve Serum (FCS) | Bioconcept | Cat# 2-01F10-I |
| Goat Serum | Sigma-Aldrich | Cat# G9023-5ML |
| **Chemicals and reagents** | | |
| Phosphate Buffered Salt Solutions (PBS) | Amimed/Bioconcept | Cat# 3-05F39 |
| RPMI 1640 | Life Technologies | Cat# 21875034 |
| 2-Mercaptoethanol | Life Technologies | Cat# 31350-010 |
| EDTA (0.5M, pH 8) | Life Technologies | Cat# AM9260G |
| Hanks Buffered Salt Solution (HBSS) | Life Technologies | Cat# 14175-053 |
| DNase I | Sigma-Aldrich | Cat# DN25-100MG |
| Collagenase I | Life Technologies | Cat# 17100017 |
| Liberase TM | Roche | Cat# 05401119001 |
| LIVE/DEAD Near IR Dead cell Stain Kit | Thermo Fisher | Cat# L10119 |
| BD Cytofix/Cytoperm reagent | BD Biosciences | Cat# 554714 |
| QuantiChrom Creatinine Assay Kit | BioAssay Systems | Cat# DICT-500 |
| QuantiChrom Urea Assay Kit | BioAssay Systems | Cat# DIUR-100 |
| Annexin V Apoptosis Detection Kit with 7-AAD | Biolegend | Cat# 640922 |
| APC BrdU Flow Kit | BD Biosciences | Cat# 552598 |
| PrimeFlow RNA Assay Kit | Thermo Fisher | Cat# 88-18005-204 |
| DAPI | Sigma-Aldrich | Cat# D9542 |
| Luminol | Sigma-Aldrich | Cat# A8511-5G |
| Cell Proliferation Reagent WST-1 | Roche | Cat# 5015944001 |
| Sytox Green | Thermo Fisher | Cat# S7020 |
| Paraformaldehyde | Sigma-Aldrich | Cat# P6148-500G |
| Percoll | Sigma-Aldrich | Cat# P1644 |
| L-Glutamine | Thermo Fisher | Cat# X0550-100 |
| Penicillin/Streptomycin | Amimed/Bioconcept | Cat# 4-01F00-H |
| O.C.T. compound | Tissue-TEK | Ref. 4583 |
| Methanol | Merck | Cat# 1.06009.2500 |
| Nonidet P40 (NP40) | Axonlab | Cat# A1694,0250 |
| Triton X-100 | BioChemica | Cat# A1388 |
| Borgal 24% | MSD Animal Health GmbH | Cat# QJ01EW13 |
| BD Calibrite Beads | BD Bioscience | Cat# 349502 |
| Yeast Extract | BD Bioscience | Cat# 212720 |
| Glucose monohydrate | Sigma-Aldrich | Cat# 49159 |
| Ampicillin | BioChemica | Cat# A08390100 |
| Bacto™ Peptone | BD Bioscience | Cat# 211820 |
| Bacto™ Tryptic Soy Broth  (Soybean-Casein Digest Medium) | BD Bioscience | Cat# 211825 |
| Malt extract | Sigma-Aldrich | Cat# 70167 |
| Glycerol (99%) | Honeywell | Cat# 10314830 |
| Ox-bile | Sigma-Aldrich | Cat# 70168 |
| Tween-40 | Sigma-Aldrich | Cat# P1504 |
| Peptone | Oxoid | Cat# LP0037 |
| Oleic Acid | Sigma-Aldrich | Cat#75090 |
| Native Olive Oil | Commerc. available | N/A |
| Agar | Sigma-Aldrich | Cat# A1296-1KG |
| **Experimental models: mouse strains** | | |
| Mouse: WT: C57BL/6JRj | Janvier Elevage | C57BL/6JRj |
| Mouse: *Il23a^-/-^* | Burkhard Becher, University of Zurich,  [58] | MGI:3625894 |
| Mouse: *Il23r*^gfp/gfp^ | Burkhard Becher, University of Zurich, [25] | MGI:3844602 |
| Mouse: *Il17ra^-/-^* | Amgen, [50] |  |
| Mouse: *Rag2^-/-^Il2rg^-/-^* | [59,60] | MGI:1858556,  MGI:3776426 |
| Mouse: *Csf2^-/-^* | Burkhard Becher, University of Zurich, [61] |  |
| **Software and algorithms** | | |
| GraphPad Prism V6 | GraphPad | *graphpad.com* |
| NDP.view2. | Hamamatsu | *hamamatsu.com* |
| FlowJo V10 | FlowJo LLC | *flowjo.com* |
| Phenochart | PerkinElmer | *perkinelmer.com* |
| inForm | PerkinElmer | *perkinelmer.com* |
| **Other** | | |
| Gallios Flow Cytometer | Beckman Coulter | beckmancoulter.com |
| FACS Aria III | BD Bioscience | bdbiosciences.com |
| Sony SP6800 Spectral Analyzer | Sony Biotechnology | sonybiotechnology.com |
| NanoZoomer 2.0-HT | Hamamatsu | *hamamatsu.com* |
| Infinite 200 plate reader | Tecan | *lifesciences.tecan.com* |
| Transpore Hypoallergic Tape | 3M | Cat# 1527-1 |
